# Limitations of transcutaneous carbon dioxide monitoring in apneic oxygenation

**DOI:** 10.1371/journal.pone.0286038

**Published:** 2023-06-01

**Authors:** Thilo Schweizer, Volker Hartwich, Thomas Riva, Heiko Kaiser, Lorenz Theiler, Robert Greif, Sabine Nabecker

**Affiliations:** 1 Department of Anaesthesiology and Pain Medicine, Inselspital, Bern University Hospital, University of Bern, Bern, Switzerland; 2 Independent Researcher, Bern, Switzerland; 3 Unit for Research and Innovation, Department of Paediatric Anaesthesia, Istituto Giannina Gaslini, Genova, Italy; 4 Centre for Anaesthesiology and Intensive Care Medicine, Hirslanden Klinik Aarau, Hirslanden Group, Aarau, Switzerland; 5 Department of Anesthesiology, Cantonal Hospital Aarau, Aarau, Switzerland; 6 School of Medicine, Sigmund Freud University Vienna, Vienna, Austria; 7 Department of Anesthesiology and Pain Management, Sinai Health System, University of Toronto, Toronto, Canada; University of Texas Medical Branch at Galveston, UNITED STATES

## Abstract

**Background:**

High-flow nasal oxygenation is increasingly used during sedation procedures and general anesthesia in apneic patients. Transcutaneous CO_2_ (p_tc_CO_2_)-monitoring is used to monitor hypercapnia. This study investigated p_tc_CO_2_-monitoring during apneic oxygenation.

**Methods:**

We included 100 patients scheduled for elective surgery under general anesthesia in this secondary analysis of a randomized controlled trial. Before surgery, we collected p_tc_CO_2_ measured by TCM4 and TCM5 monitors and arterial blood gas (ABG) measurements every two minutes during 15 minutes of apnea. Bland-Altman plots analyzed agreement between measurement slopes; linear mixed models estimated the different measuring method effect, and outlined differences in slope and offset between transcutaneous and arterial CO_2_ partial pressures.

**Results:**

Bland-Altman plots showed a bias in slope (95% confidence intervals) between ABG and TCM4-measurements of 0.05mmHg/min (-0.05 to 0.15), and limits of agreement were -0.88mmHg/min (-1.06 to -0.70) and 0.98mmHg/min (0.81 to 1.16). Bias between ABG and TCM5 was -0.14mmHg/min (-0.23 to -0.04), and limits of agreement were -0.98mmHg/min (-1.14 to -0.83) and 0.71mmHg/min (0.55 to 0.87). A linear mixed model (predicting the CO_2_-values) showed an offset between arterial and transcutaneous measurements of TCM4 (-15.2mmHg, 95%CI: -16.3 to -14.2) and TCM5 (-19.1mmHg, -20.1 to -18.0). Differences between the two transcutaneous measurements were statistically significant.

**Conclusions:**

Substantial differences were found between the two transcutaneous measurement systems, and between them and ABG. Transcutaneous CO_2_ monitoring cannot replace arterial CO_2_-monitoring during apneic oxygenation. In clinical settings with rapidly changing CO_2_-values, arterial blood gas measurements are needed to reliably assess the CO_2_-partial pressure in blood.

**Trial registration:**

ClinicalTrials.gov (NCT03478774).

## Introduction

High-flow nasal oxygenation (HFNO) delivers heated, humidified oxygen via nasal cannulas at high rates of gas flow [1]. It is used for procedural interventions during spontaneous ventilation and during apneic oxygenation under general anesthesia [2–4]. The factor limiting the use of HFNO is the increase in carbon dioxide (CO_2_), not the drop in blood oxygen level [5, 6]. During apnea, oxygenation is maintained by a constant influx of 100% oxygen into the lungs, but elimination of CO_2_ is insufficient [7]. While monitoring of end-tidal carbon dioxide (p_et_CO_2_) from the expiratory arm of the breathing circuit via capnography is standard care during anesthesia in ventilated patients, it cannot be used during HFNO in apneic patients due to the open system.

Following its successful implementation in neonatology, pediatrics, pulmonology and sleep medicine, transcutaneous CO_2_ (p_tc_CO_2_) monitoring is increasingly used in adult anesthesia and critical care [8–10]. Using transcutaneous CO_2_ for non-invasive monitoring of carbon dioxide seems suitable in apneic patients receiving HFNO [11]. However, there is not yet agreement how accurate these measurements in reality are, as p_tc_CO_2_ and p_et_CO_2_ did not correlate in one out of three patients [12].

Currently, there is lack of evidence about the quality of p_tc_CO_2_ measurements in anesthetized apneic patients with rapidly changing arterial partial pressures of CO_2_ (p_a_CO_2_).

### Outcomes

This secondary analysis of a randomized controlled trial published before [13, 14] tested the hypothesis that the slope of p_tc_CO_2_ measured with two similar transcutaneous p_tc_CO_2_ measurement monitors adequately reflects the arterial CO_2_ ascent during nasal oxygen for apneic oxygenation under general anesthesia. Therefore, the primary outcome was the transcutaneous CO_2_ measurements obtained from the transcutaneous CO_2_ monitors TCM4 and TCM5.

## Methods

The Cantonal Ethics Committee of Bern approved the study (2018–00293), which was registered at ClinicalTrials.gov (NCT03478774) and the Swiss Trial Registry KOFAM (SNCTP000002861).

### Trial design

This manuscript reports a side protocol of a randomized controlled non-inferiority trial and was carried out according to the STROBE statement [13, 14].

### Participants, eligibility criteria and settings

The detailed study intervention and inclusion and exclusion criteria were published previously [14]. Operating room lists were screened for eligible patients daily. Included were adult patients younger than 80 years with an American Society of Anesthesiologists (ASA) physical status of I to III. Excluded were patients with expected difficult mask ventilation, obstructive sleep apnea syndrome requiring therapy, necessity of rapid sequence induction due to aspiration risk, known chronic obstructive pulmonary disease GOLD classification 2 or higher, cervical spine instability, the need for flexible optic intubation, nasal obstruction, pregnancy, known coronary heart disease, arrythmias requiring therapy, known heart failure higher than New York Heart Association classification I, treatment with beta-receptor antagonists, known stenosis of the carotid or vertebral arteries, peripheral arterial disease with a Fontaine classification higher than 2b, hyperkalemia (potassium level higher than 5.5 mmol/l), pulmonary arterial hypertension with an pressure higher than 35mmHg, body mass index of less than 16 kg/m^2^ or more than 35 kg/m^2^, known increased intracranial pressure, patients scheduled for intracranial surgery, anemia with hemoglobin less than 100g/l, known muscular disorders and allergies or contraindications to the anesthesia agents used in the study [13, 14].

Written informed consent was obtained from all study participants, and the study was conducted according to the guidelines of the Declaration of Helsinki. Data was obtained at the Bern University Hospital in Bern, Switzerland, as part of an investigation into a possible ventilatory effect of different flow rates of trans nasal oxygen delivery between March 2018 and December 2019.

### Interventions

Anesthetized patients under neuromuscular blockade were evaluated during apnea over a 15-minute period before elective surgery. During this study, CO_2_ partial pressure was measured continuously via transcutaneous monitors and in arterial blood gases every 2 minutes [14].

We used TCM4 and TCM5, two transcutaneous CO_2_ monitoring devices produced by the same manufacturer (Radiometer, Krefeld, Germany). Both are using the same measurement algorithm. The TCM sensors were placed as recommended by the manufacturer in the sub clavicular area on the patients’ chest. The probe temperature of the TCM4 was set at 44°C for all measurements. The probe temperature of the TCM5 was set at 44°C for the first 39 patients and at 42°C in the 57 following patients to compare a possible effect of temperature on the measurement accuracy. With both monitors, the Sensor 84 (Radiometer, Krefeld, Germany) was used to determine O_2_ and CO_2_ partial pressures at the same time. To differentiate between the combined O_2_/CO_2_ sensor and a CO_2_-only sensor, we used the Sensor 54 (Radiometer, Krefeld, Germany) with the TCM5 in five patients as a control.

The gold standard reference method for measuring CO_2_ partial pressure is arterial blood gas (ABG) analysis [15]. Arterial blood samples were drawn every 2 minutes using a safePICO syringe (Radiometer, Krefeld, Germany) and were analyzed with an ABL800 flex blood gas analyser (Radiometer, Krefeld, Germany) according to the manufacturer’s manual at the certified central laboratory of the Bern University Hospital.

After arrival of the patient in the operating theatre, the probes of the two TCM monitors were applied on the patient’s chest and an arterial Flowswitch cannula (BD, Franklin Lakes, USA) was placed ultrasound-guided into the radial artery to measure blood pressure and to draw arterial blood samples. Induction and maintenance of general anesthesia were standardized as described earlier [14]. After induction of anesthesia with neuromuscular blockade and successful bag-mask ventilation, apneic oxygenation was commenced as randomized. Four groups received oxygen via nasal cannulas. One group received 2 l/min O_2_ via standard nasal cannula and the airway was continuously kept patent applying jaw thrust. Another group received 10 l/min O_2_ via standard nasal cannula plus jaw thrust. Two groups received 70 l/min O_2_ via a high-flow nasal cannula, the airway was kept patent in one of them with jaw trust, and in the other group with a video laryngoscope. The fifth group received 0.25 l/min O_2_ via a tracheal tube, therefore, we did not include this group into the current analysis, as only apneic oxygenation via nasal cannula was within the scope of this sub-study [14]. Absence of muscular twitch during electrical stimulation showed complete neuromuscular blockade (TOF-Watch; Organon Ltd, Dublin, Ireland). Absence of diaphragmatic movements was assessed with continuous electrical impedance tomography (PulmoVista 500; Draeger, Luebeck, Germany). Norepinephrine was used to maintain normotension, which was defined as values within 20% of pre-operative values measured on the ward before surgery.

Before start of anesthesia, and again from the start of apneic oxygenation, arterial blood gas samples were drawn every two minutes and p_tc_CO_2_ values were recorded every minute. After completion of the study, airway management and surgery were performed as planned.

### Sample size

Sample size calculation was performed for the main study, where a difference between p_a_CO_2_ group means of 0.3mmHg/min was assumed as clinically relevant, using a two-sample t-test, assuming a non-inferiority margin of 0.3, a common standard deviation of 0.35, a power of 80% and a one-sided alpha of 0.025. Twenty-five patients were found to be necessary per group (including 3 patients per group as a safety margin) [14].

### Randomization

Patients were stratified according to body mass index and smoking status, then randomly allocated to one of the five study groups. Computer-generated randomization (www.randomisation.com) was used by the research staff of the department to allocate study participants to the respective groups and was kept in sealed opaque envelopes, which were opened after induction of anesthesia and successful bag-mask ventilation by a study nurse not involved in the randomization process.

### Blinding

As patients were under anaesthesia they were blinded to their allocated group, but due to the different delivery of oxygen and the different procedures to keep the airway open blinding the study staff was not possible [14].

### Interim analysis and stopping rules

There was no interim analysis planned and performed. The predefined clinical stopping rules for study patients were S_p_O_2_ less than 92%, transcutaneous carbon dioxide greater than 100 mmHg, pH less than 7.1, potassium greater than 6 mmol l^–1^, or an apneic period of 15 minutes. Reaching one of these criteria terminated the apnea, and bag-mask ventilation was started.

### Statistical analysis

**Fig 1:**
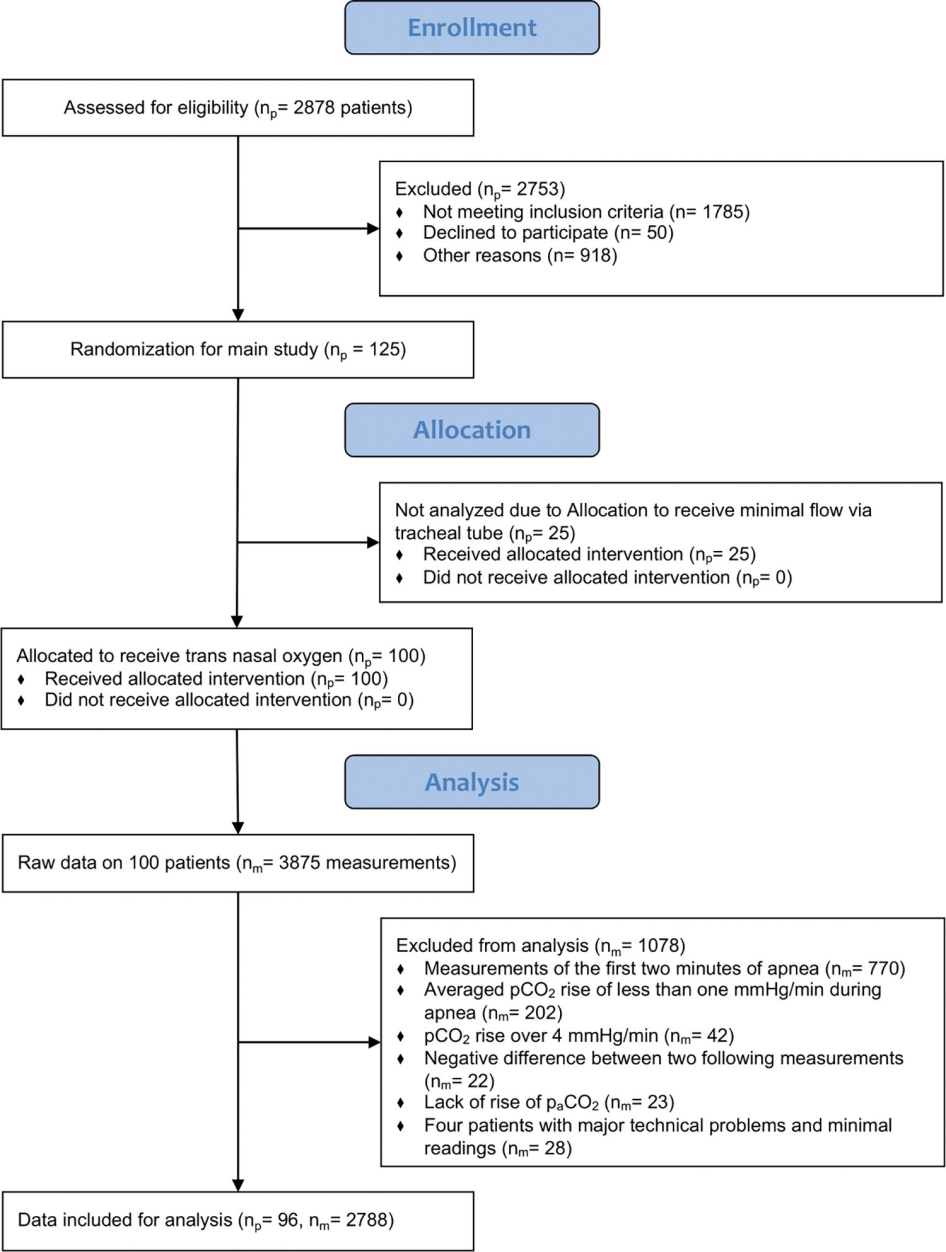
Modified CONSORT flow diagram with numbers of patients (n_p_) and numbers of measurements (n_m_).

All recorded study data were transferred into an electronic database (REDCap, Research Electronic Data Capture, Vanderbilt University, 2004). There were no differences of increase in p_a_CO_2_ detected between flow rate groups in the main study, therefore we analysed the groups together [14] We defined filter criteria for the data to transparently exclude technical errors and other biases. For example, an averaged p_tc_CO_2_ increase of less than one mmHg/min during the apneic period would reflect insufficient attachment of the transcutaneous sensor or lack of a proper seal against CO_2_ volatilization into room air (for transparency, S1 Appendix displays statistics and graphs based on the unfiltered raw data). In the first minutes of apnea, p_a_CO_2_ increases faster than afterwards, because CO_2_ in arterial and venous blood is equilibrating. As a result, measurements of the first three apneic minutes were excluded from analysis (Fig 1) [16]. According to the literature, an increase in CO_2_ of more than four mmHg/min after the first minutes of apnea in anesthetized patients under neuromuscular blockade is unlikely, therefore we excluded higher values [7, 16]. We also excluded transcutaneous measured values if they were lower than the measurements one minute before, or ABG measurements if they were lower or equal to the previous results. Such lower readings are not plausible during apneic oxygenation and were considered to be technical errors or sampling dilution.

We used Bland-Altmann plots (presenting mean slopes, mean difference in slopes and 95% limits of agreement) to test our hypothesis, the agreement between the increase of p_a_CO_2_ and p_tc_CO_2_ over time [17]. Linear mixed models were fitted to the data in order to estimate the effect of the measuring method on CO_2_ values and to outline differences in slope and offset between the two transcutaneous monitors. Method, time, and their interaction were used as fixed covariates, together with random intercepts and slopes for patients. The models were fitted with restricted maximum likelihood and 95% confidence intervals (CI), and p-values were calculated using Satterthwaite’s approximation for the degrees of freedom. Normality of residuals and random effects were assessed visually using Q-Q-plots; variance homogeneity using residuals-vs-fitted plots. Subgroup analysis was performed for sensor temperature.

Results are presented as numbers (%) or mean ± SD. A p-value <0.05 was considered statistically significant. Analyses were performed using Stata version 16.1 (StataCorp LT, Texas, USA) or R version 4.0.3. (R Core Team (2020), R Foundation for Statistical Computing, Vienna, Austria).

## Results

The CONSORT flow diagram is displayed as Fig 1. One hundred twenty-five patients were enrolled in the main study, 100 received nasal oxygen and were included in this secondary analysis [14]. Measurements of four patients were excluded due to major technical problems. (Fig 1). Patients’ characteristics are displayed in Table 1.

**Table 1:** Patients’ characteristics.

Characteristic	n = 96
Age (years)	48 ± 18
Gender (male)	49 (51)
BMI (kg/m^2^)	25 ± 4
ASA (I/II/III)	21/67/8 (22/70/8)
Orthopedic surgery	56 (58)
Visceral surgery	31 (32)
Thoracic surgery	6 (6)
Neurosurgery	3 (3)

Data are numbers (%) or mean ± SD

**Fig 2:**
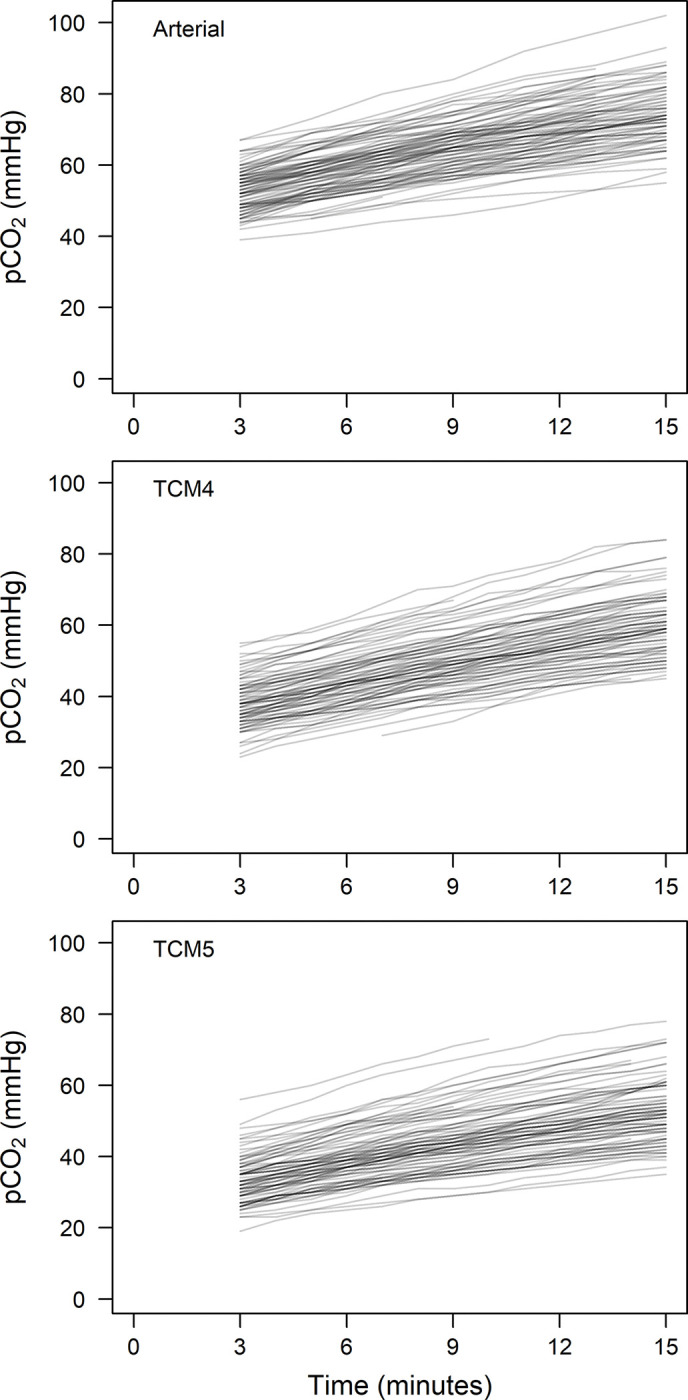
Measurements of CO_2_ over time for each patient using the two transcutaneous methods (TCM4 and TCM5) and arterial blood gas analysis.

Fig 2 depicts the graphical analysis of all CO_2_ measurements during the observation period.

**Fig 3:**
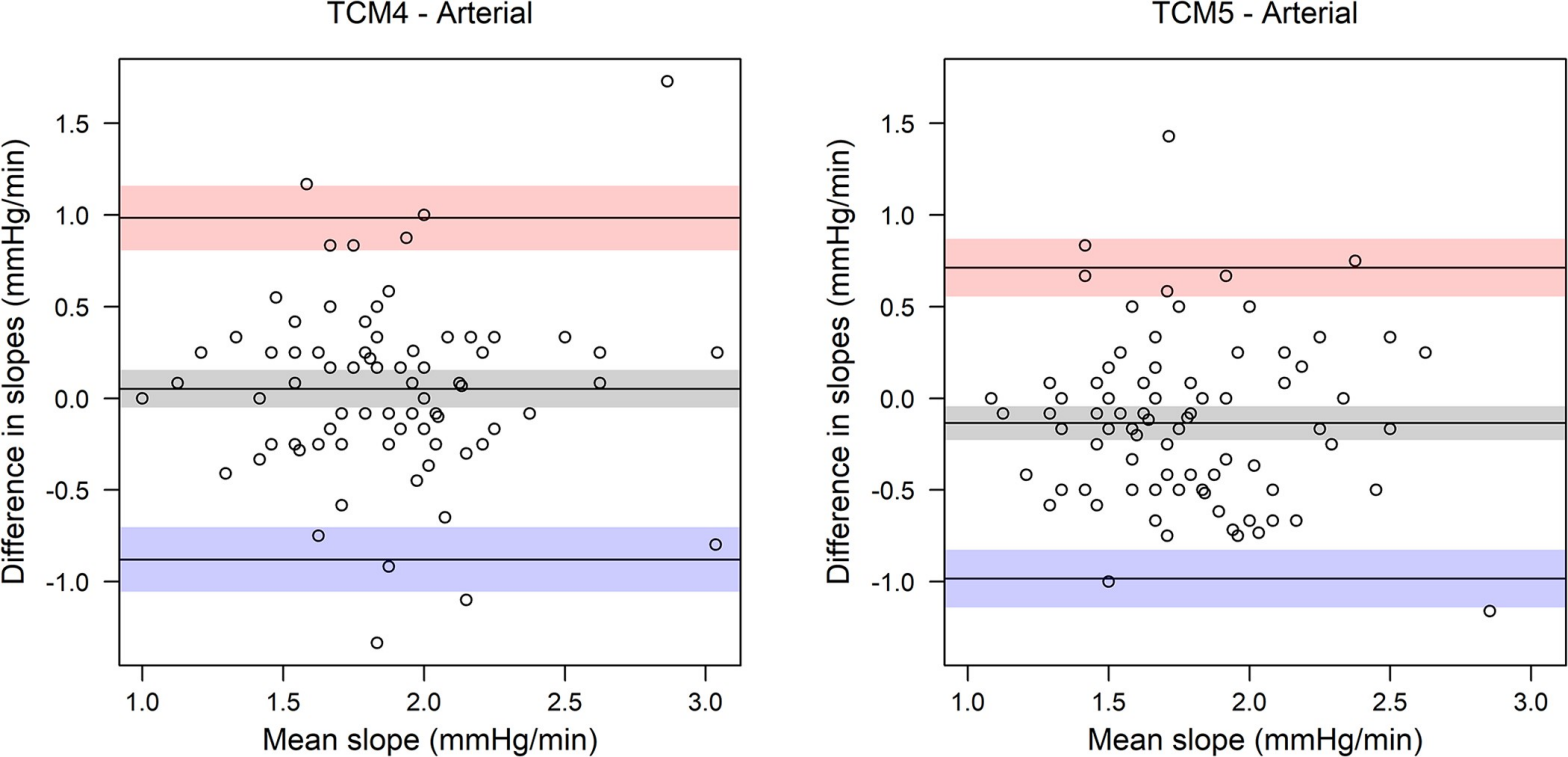
Bland-Altman plots for the two transcutaneous measurements (TCM4 and TCM5) vs. the arterial blood gas measurement using the total slope (i.e., the change per minute in CO_2_ from first to last measurement). A positive difference indicates a steeper slope for the transcutaneous measurement. The colored area indicates 95% confidence intervals for bias (grey) and limits of agreement (red, blue).

Fig 3 shows the Bland-Altman plots of the total slopes (i.e., the change per minute in CO_2_ from first to last measurement) of the two transcutaneous measurements compared to the arterial measurements. Bias in slopes (95% CI) between the ABG measurements and the TCM4 measurements were 0.05 mmHg/min (-0.05 to 0.15), and limits of agreement were -0.88 mmHg/min (-1.06 to -0.70) and 0.98 mmHg/min (0.81 to 1.16). Bias between ABG measurements and TCM5 measurements were -0.14 mmHg/min (-0.23 to -0.04), and limits of agreement were -0.98 mmHg/min (-1.14 to -0.83) and 0.71 mmHg/min (0.55 to 0.87).

**Fig 4:**
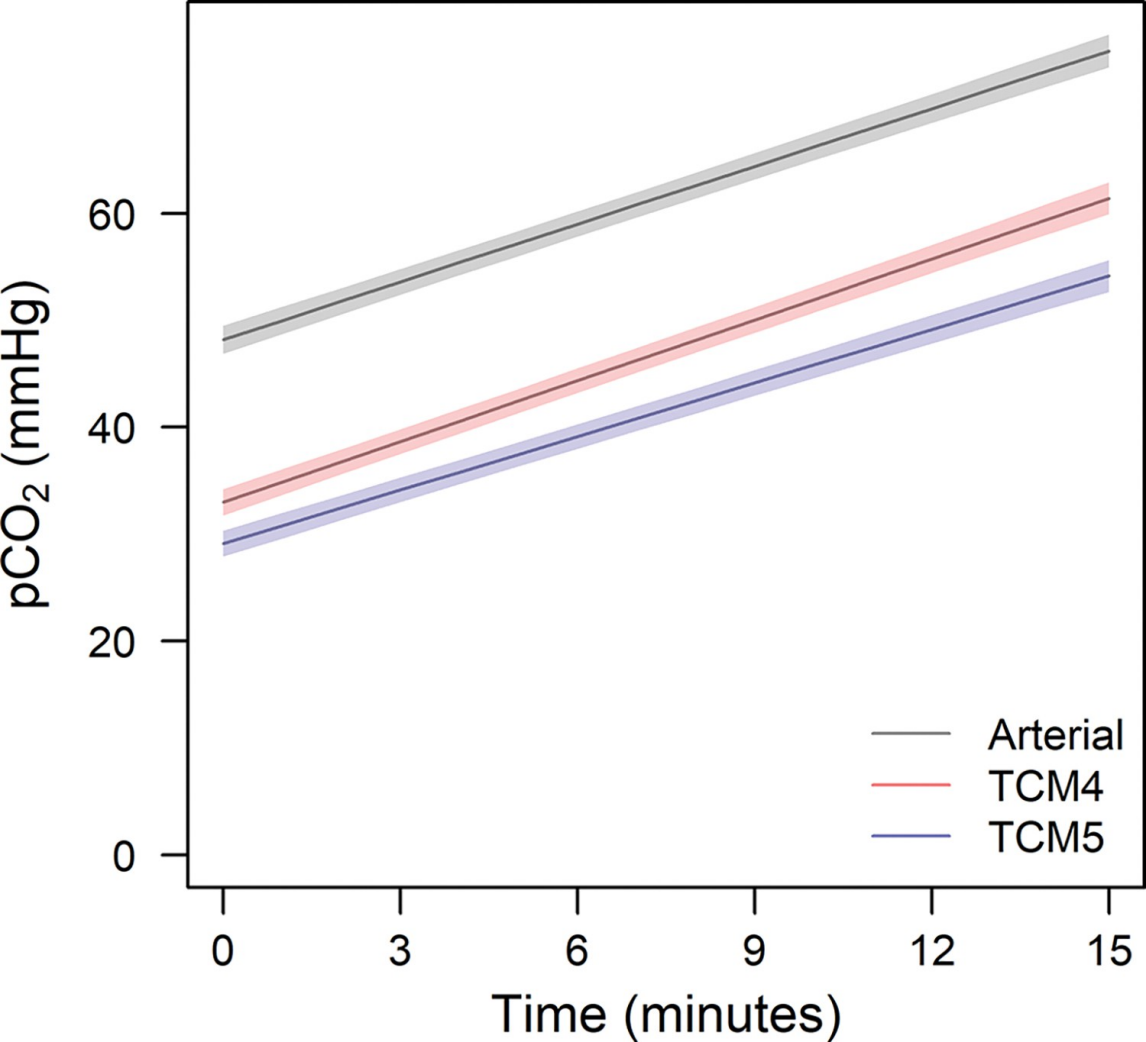
Predicted CO_2_ values with 95% confidence intervals based on the linear mixed model, including the measurements *method*, *time* and their *interaction* as fixed covariates. The offset between ABG and TCM4, ABG and TCM5, and TCM5 and TCM4 was significant, as was the difference in slope between ABG and TCM5, and TCM5 and TCM4.

**Fig 5:**
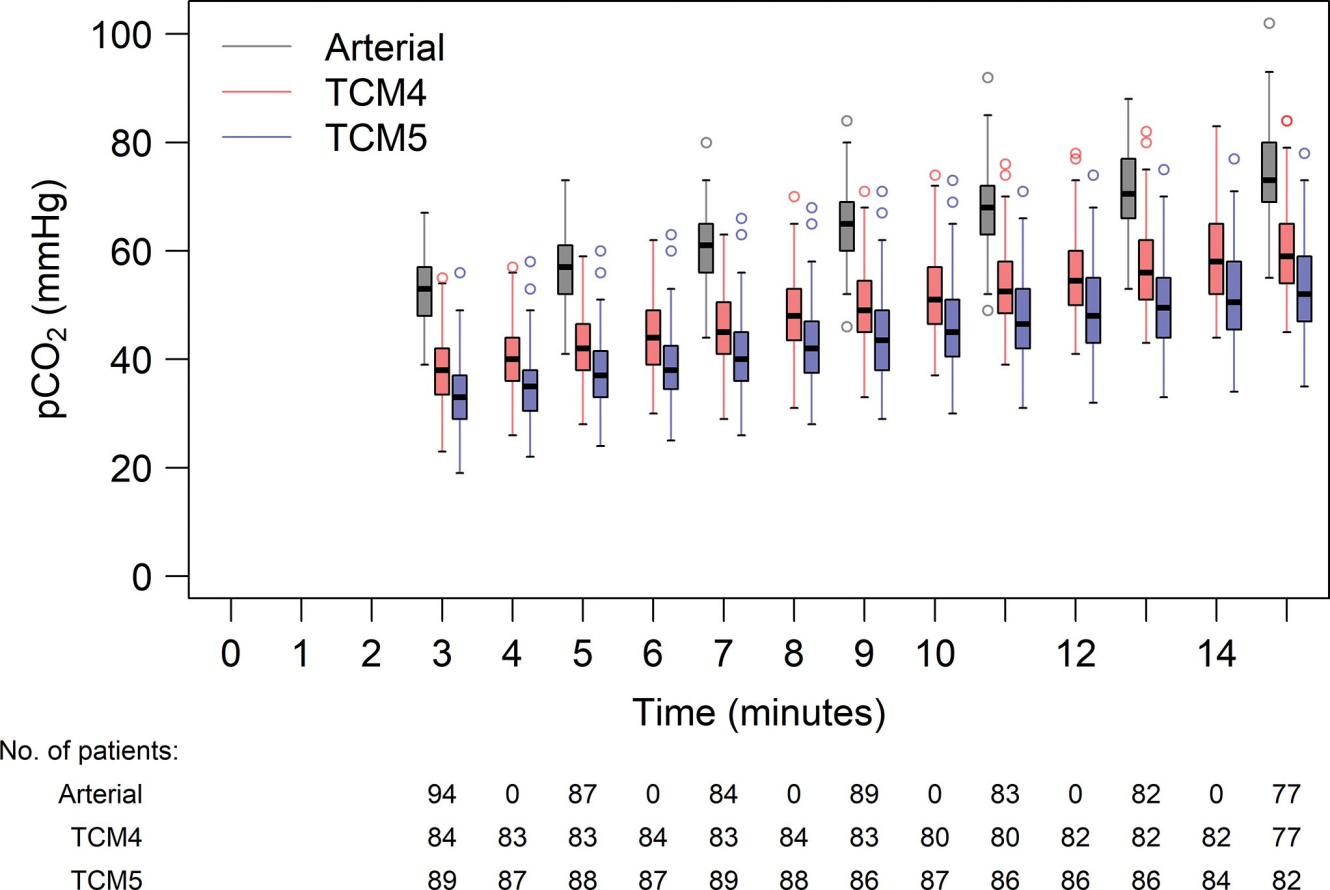
Box plots of CO_2_ measurements at each time point using the two transcutaneous methods (TCM4 and TCM5) and arterial blood gas analysis. Boxes indicate lower to upper quartiles and whiskers show the most extreme point within 1.5 times the interquartile range from the upper and lower quartile, respectively. Points beyond that range are indicated with circles.

Table 2. shows the model coefficients from a linear mixed model for CO_2_. The offset between ABG and TCM4, ABG and TCM5, and TCM5 and TCM4 was significant, as were the differences in slope between ABG and TCM5, and TCM5 and TCM4 (Table 2). This is also displayed in Figs 4 and 5 shows box plots of CO_2_ measurements every minute using the two transcutaneous monitors and ABG.

**Table 2:** Model coefficients based on the linear mixed model, mean pCO_2_ difference in offset at time 0.

	**Mean pCO**_**2**_ **difference in offset in mmHg (95%CI)**	**p-value**
ABG (Reference)	48.2 (46.9 to 49.5)	
ABG to TCM4	-15.2 (-16.3 to -14.2)	<0.001
ABG to TCM5	-19.1 (-20.1 to -18.0)	<0.001
TCM5 to TCM4	-3.9 (-4.8 to -2.9)	<0.001
	**Mean difference in slope in mmHg/min (95%CI)**	**p-value**
ABG (Reference)	1.80 (1.69 to 1.91)	
ABG to TCM4	0.10 (-0.01 to 0.21)	0.09
ABG to TCM5	-0.13 (-0.24 to -0.02)	0.018
TCM5 to TCM4	-0.23 (-0.32 to -0.13)	<0.001

ABG = arterial blood gas, TCM = transcutaneous monitor

Comparison of the different sensor temperatures (44°C and 42°C) showed no significant difference in slopes (0.07 mmHg/min (-0.08 to 0.23, p = 0.36)).

The offset of the measurements with the Sensor 54 (CO_2_-only sensor) to the ABG as reference method was -10.0mmHg (-12.2 to -7.7, p<0.001). The mean difference in slope for ABG measurements was 0.20 mmHg/min (-0.02 to 0.43, p = 0.08). An interaction model showed a difference between TCM5 measurements with the Sensor 84 (combined O_2_/CO_2_ sensor) to the measurements with the Sensor 54 of 9.3mmHg (6.2 to 12.5) in offset and 0.39 mmHg/min (0.07 to 0.71) in slope.

## Discussion

We found substantial differences between transcutaneous and arterial CO_2_ measurements, as well as between the two transcutaneous monitors investigated. From a clinical perspective, the slope of increase in p_a_CO_2_ can be regarded as linear after the initial fast increase in anesthetized patients with complete neuromuscular blockade.

So far, the dynamics of the CO_2_ increase in anesthetized, paralyzed, and apneic, but oxygenated humans are not entirely understood. Well documented is the initial fast increase in p_a_CO_2_ in the first minute, due to the equilibration of arterial and venous pCO_2_ as a result of hyperoxic apnea when minimal CO_2_ is exhaled [18]. The subsequent increase is less clear. Research showed that the total increase of p_a_CO_2_ in patients with a blocked airway can best be described as a logarithmic function, but also simplified as a piecewise linear model separating the first minute of apnea from the time following it [19]. Researchers demonstrated in five patients that the initial fast increase is followed by an almost linear increase in CO_2_ [20]. Figs 2 and 5 support the assumption that the increase of p_a_CO_2_ after the equilibration of arterial and venous pCO_2_ may be regarded as linear within the apneic period of 15 minutes. Of note, we ensured maintenance of arterial blood pressure, and the deep anesthetic state possibly counteracted sympathetic stimulation caused by rising CO_2_ levels. Previous research showed a larger flattening, which could be caused by the diminishing neuromuscular blockade over time, with consecutive small movements of the diaphragm and thereby a ventilatory effect [21].

Although the bias shown in the Bland-Altman plots in Fig 3 lies close to 0, limits of agreements differ substantially. Previous studies defined a maximum difference of 7.5 mmHg between pCO_2_ measurements as clinically acceptable in a steady-state measurement [22, 23]. If this maximum difference is also acknowledged in measurements with rising pCO_2_ after 15 minutes (regardless of the offset), limits of agreement in slope have to be within 7.5mmHg/15min, which is equal to ±0.5mmHg/min, and this seems to be clinically acceptable. The larger levels of agreement and the number of measurements outside this range indicate that the measurement techniques cannot be used interchangeably in this population and setting (Fig 3). Further research is required to understand why in some patients the slope led to a difference of more than 7.5mmHg over 15min.

Researchers tested the accuracy of p_tc_CO_2_ in patients admitted to the intensive care unit and found mean p_tc_CO_2_ to be 2.2mmHg higher than p_a_CO_2_ (limits of agreement: -9.2 to 13.6mmHg) [24]. In contrast, in our standardized anesthesia setting, we found a far greater difference in offset at time 0 (-15.2 mmHg between ABG and TCM4, and -19.1 mmHg between ABG and TCM5). One hypothesis for the reasons for these differences is that the sensors provided by the manufacturers were probably very new in these studies [9, 22–24]. Response time of transcutaneous CO_2_ electrodes lies usually between 20-80s, but with increasing age, it slows down over time [25, 26]. This might play a more important role during the monitoring of CO_2_ in apneic oxygenation with fast-changing CO_2_ values rather than in steady-state measurements in the intensive care setting [27]. Unfortunately, manufacturers do not provide information on the sensors’ time-function association.

Reliability can be limited by some technical pitfalls that can lead to inaccurate measurements: trapped air bubbles between the skin and the sensor, incorrect sensor placement, and incorrect sensor maintenance [15]. In addition, a propofol-induced peripheral vasodilatation-mediated drop in core body temperature might lead to hypothermia which could impair trending ability [28].

It is not completely clear why the CO_2_-only sensor (sensor 54) showed a smaller offset to the ABGA measurements than the combined O_2_/CO_2_ sensor (sensor 84). Due to the small number of measurements performed with the sensor 54, this result should be re-evaluated in future studies.

In clinical practice, it might be useful to compare transcutaneous CO_2_ values with an arterial CO_2_ measurement at the beginning of apnea in order to determine the offset. Subsequent arterial CO_2_-measurements might then be used to determine the slope, which would improve the reliability on which clinical decisions could be made. Therefore, we recommend an arterial blood gas analysis at the beginning of apnea, and further blood gas analysis every 10 minutes later to allow for better therapeutic decisions under apneic oxygenation.

In healthy patients without pre-existing diseases, transcutaneous CO_2_ measurements might be suitable for CO_2_ trend monitoring. In those patients, comparison with p_et_CO_2_ prior to apnea might be sufficient. However, arterial blood gas analysis should be performed if there is uncertainty about the presence of hypercarbia to ensure patient safety.

Potential limitations of our study are the single-centre study design and the fact that all measurements were made in apneic patients of our specific patient population (compare inclusion/exclusion criteria). Further studies of other users in different settings and including different patient populations are needed to confirm and generalize our observations and their clinical implications. As our observation period was only 15 minutes, we cannot make conclusive statements for longer periods of time and if these devices might be used as trend monitors. Approximately 7.3% of data measured after the first minutes had to be excluded from the analysis (based on previously published data) to verify correct measurements, e.g., a rise of CO_2_ between 1-4mmHg/min [7]. We cannot comment on why this happened and if more reliable CO_2_ measurements would have provided different results.

## Conclusions

In conclusion, transcutaneous CO_2_ monitoring cannot replace arterial CO_2_ measurements during apneic oxygenation as commercially available transcutaneous CO_2_ monitors showed inconsistent deviations from the gold standard, which remains arterial blood gas analysis. The offset between arterial CO_2_ partial pressure and the transcutaneous CO_2_ partial pressure measurements, as well as the increase in the CO_2_ slopes varied significantly over time.

## Supporting information

S1 Checklist CONSORT 2010 checklist of information to include when reporting a randomised trial*. (DOC)

S1 Appendix Statistics and graphs based on the unfiltered data. (PDF)

S1 Protocol (DOCX)
