# Supplementary material for: Limitations of transcutaneous carbon dioxide monitoring in apneic oxygenation
Source: PLoS One. 2023 Jun 1;18(6):e0286038. doi: 10.1371/journal.pone.0286038 (PMC10234524; doi:10.1371/journal.pone.0286038)
Supplement: S1 Appendix — (PDF) [file pone.0286038.s002.pdf]

S1 Appendix. Statistics and graphs based on the unfiltered data.

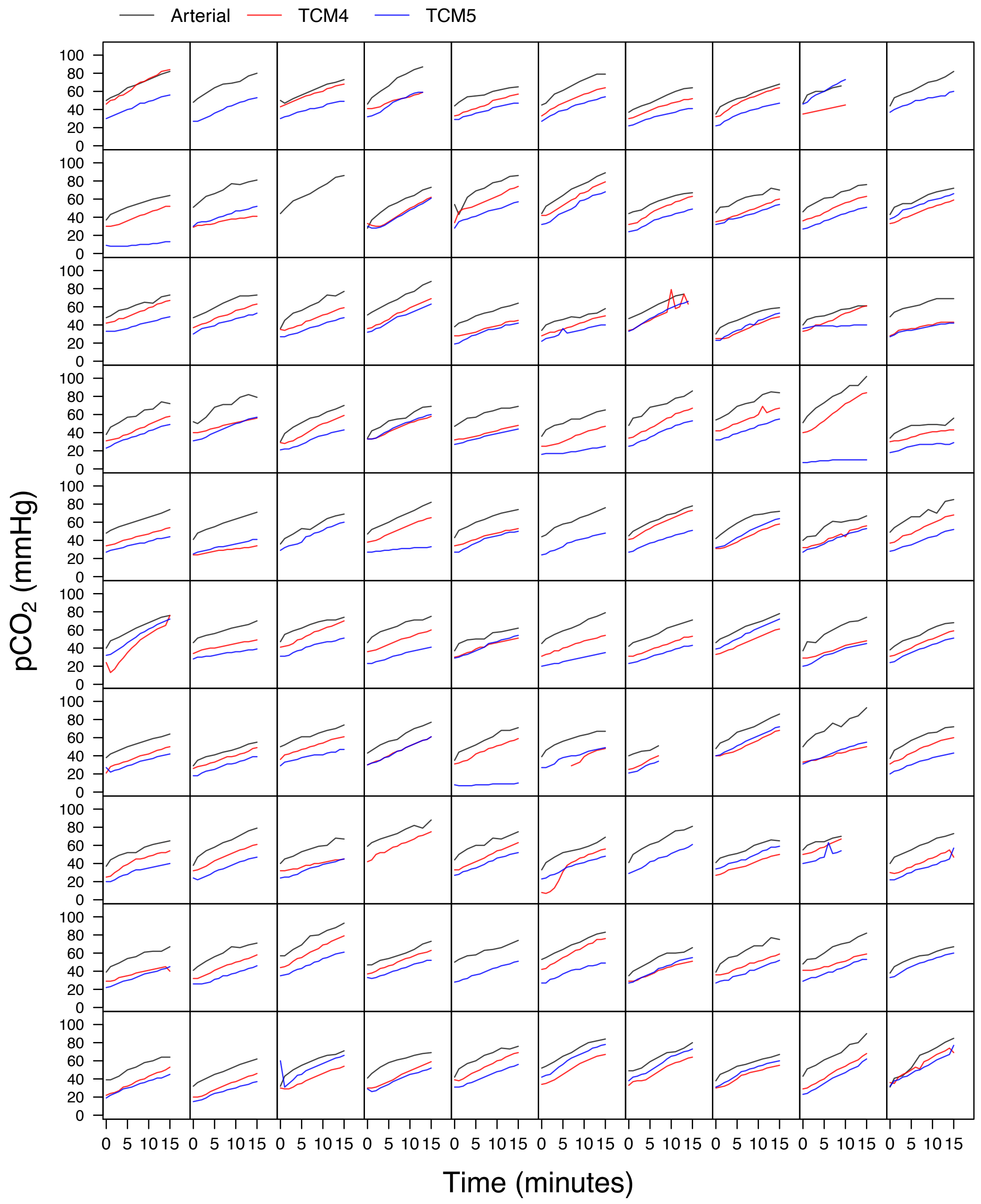

Figure S1. Measurements of pCO_2 over time for each patient using the two transcutaneous methods (TCM4 and TCM5, respectively) and arterial blood gas analysis based on the unfiltered data.

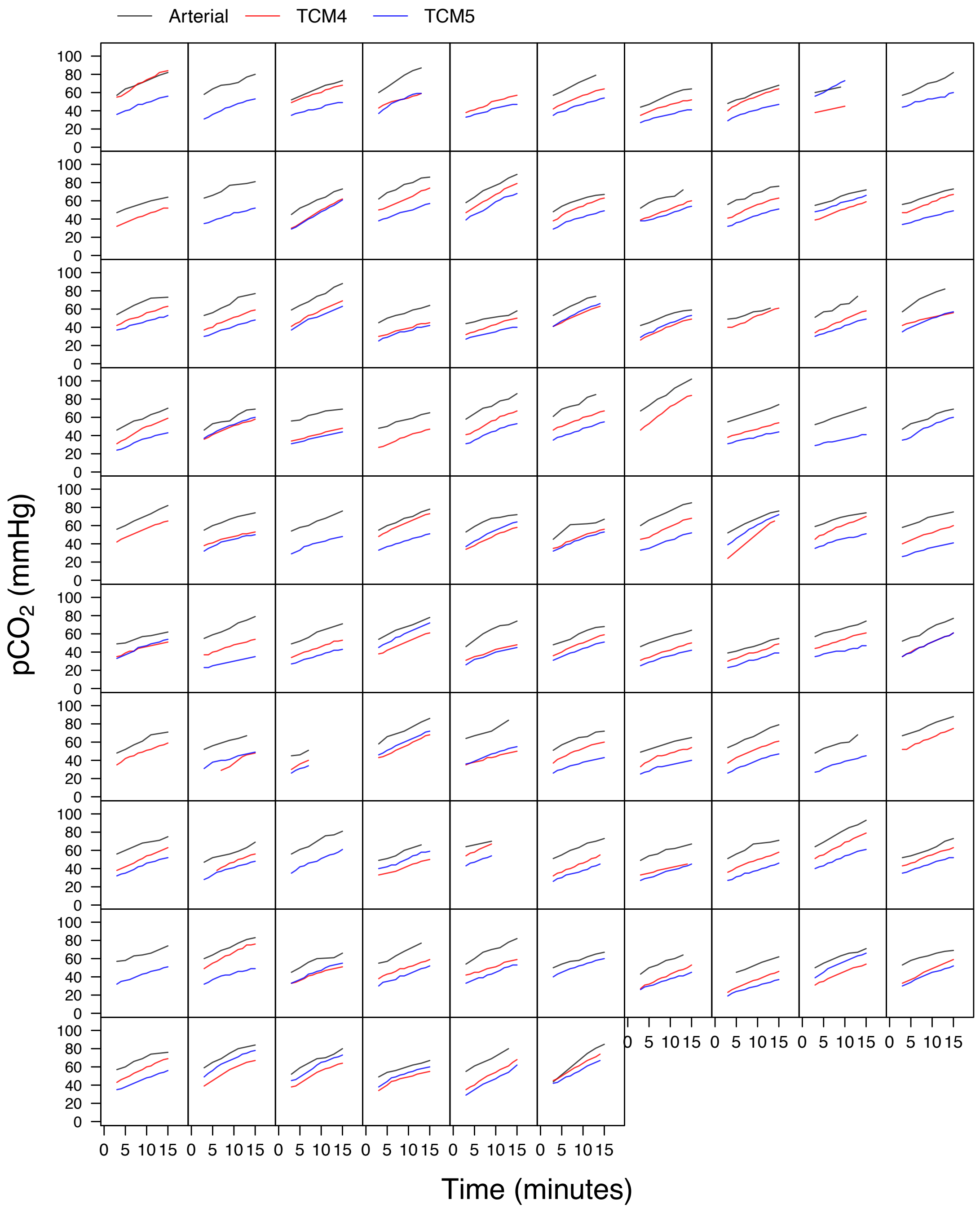

Figure S2. Measurements of pCO_2 over time for each patient using the two transcutaneous methods (TCM4 and TCM5, respectively) and arterial blood gas analysis based on the filtered data.

Table S2. Model coefficients based on the linear mixed model, mean pCO_2 difference in offset at time 0 based on the unfiltered data. ABG = arterial blood gas, TCM = transcutaneous monitor

	**Mean pCO_2 difference in offset in mmHg (95%CI)**	**p-value**
ABG (Reference)	45.7 (44.5 to 46.9)	
ABG to TCM4	-13.2 (-14.0 to -12.3)	<0.001
ABG to TCM5	-17.9 (-18.7 to -17.0)	<0.001
TCM5 to TCM4	-4.7 (-5.46 to -3.94)	<0.001
	**Mean difference in slope in mmHg/min (95%CI)**	**p-value**
ABG (Reference)	2.03 (1.92 to 2.13)	
ABG to TCM4	-0.15 (-0.24 to -0.05)	0.002
ABG to TCM5	-0.42 (-0.51 to -0.32)	<0.001
TCM5 to TCM4	-0.27 (-0.36 to -0.18)	<0.001

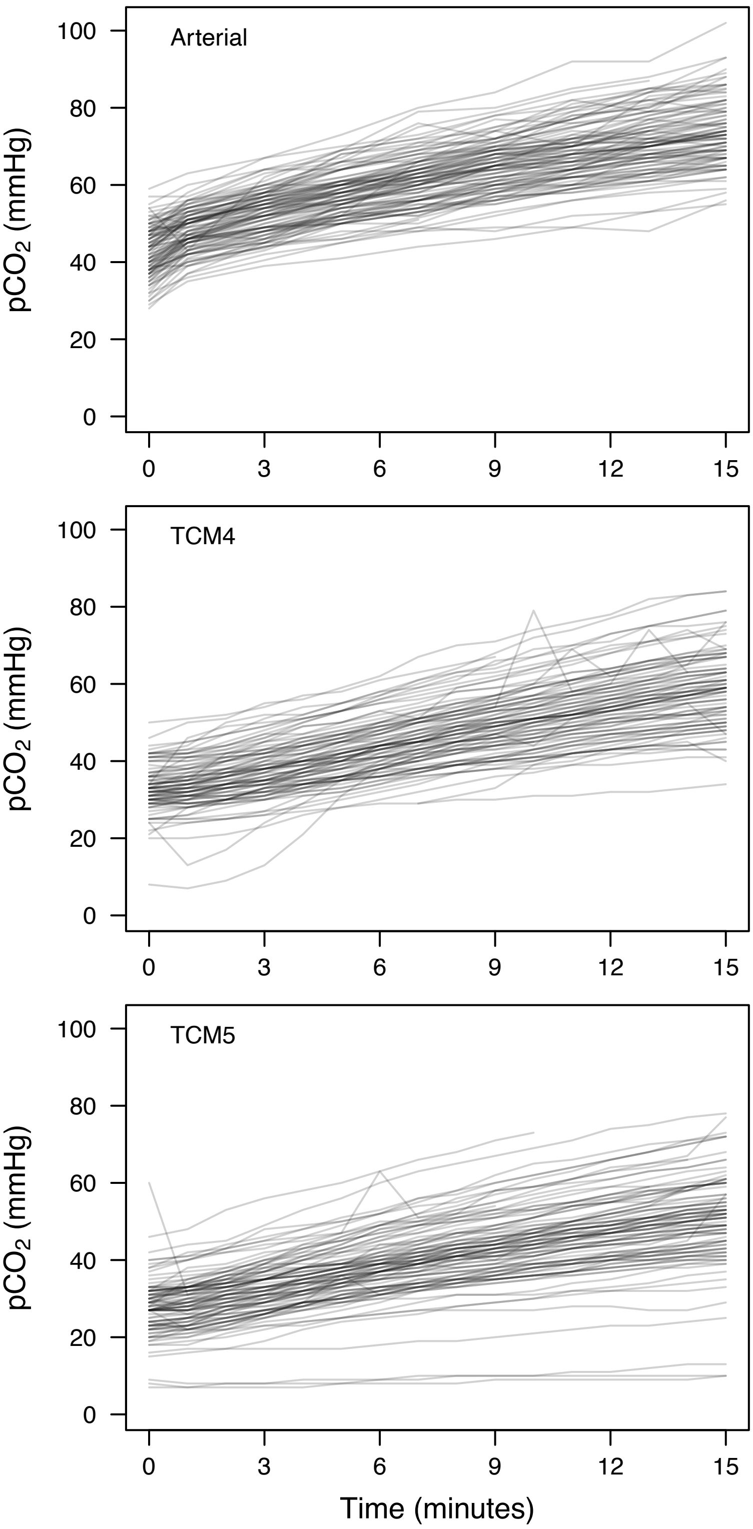

Figure S2. Measurements of CO2 over time for each patient using the two transcutaneous methods (TCM4 and TCM5) and arterial blood gas analysis based on the unfiltered data.

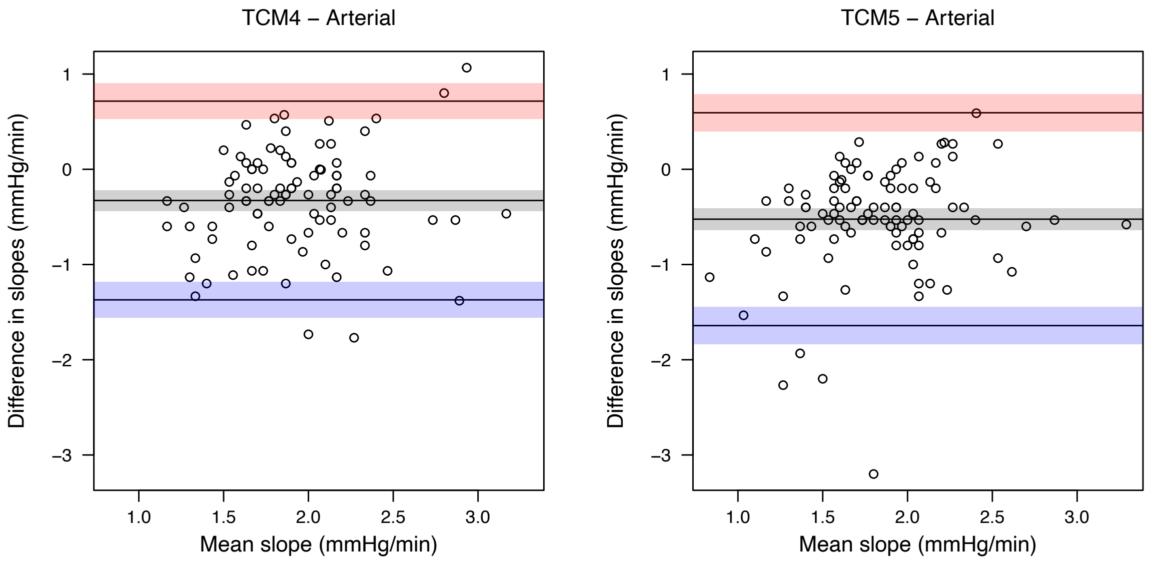

Figure S3. Bland-Altman plots for the two transcutaneous measurements (TCM4 and TCM5) vs. the arterial blood gas measurement using the total slope (i.e., the change per minute in CO2 from first to last measurement) based on the unfiltered data. A positive difference indicates a steeper slope for the transcutaneous measurement. The colored area indicates 95% confidence intervals for bias (grey) and limits of agreement (red, blue).

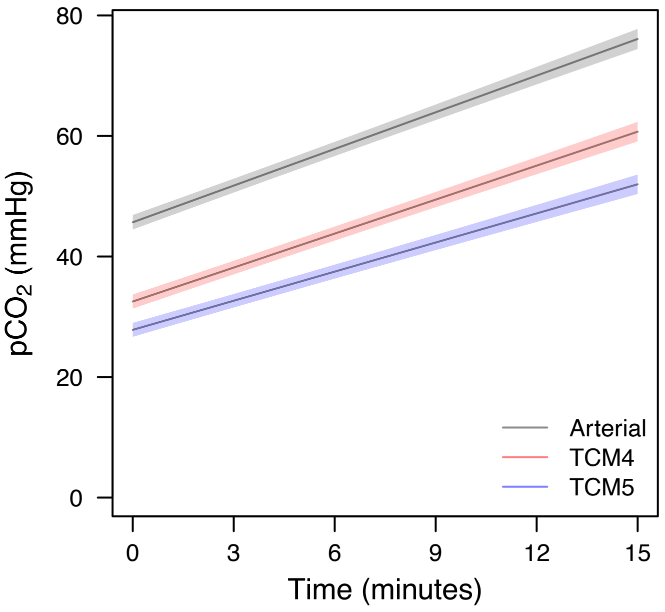

Figure S4. Predicted CO2 values with 95% confidence intervals based on the linear mixed model of the unfiltered data, including the measurements *method*, *time* and their *interaction* as fixed covariates.

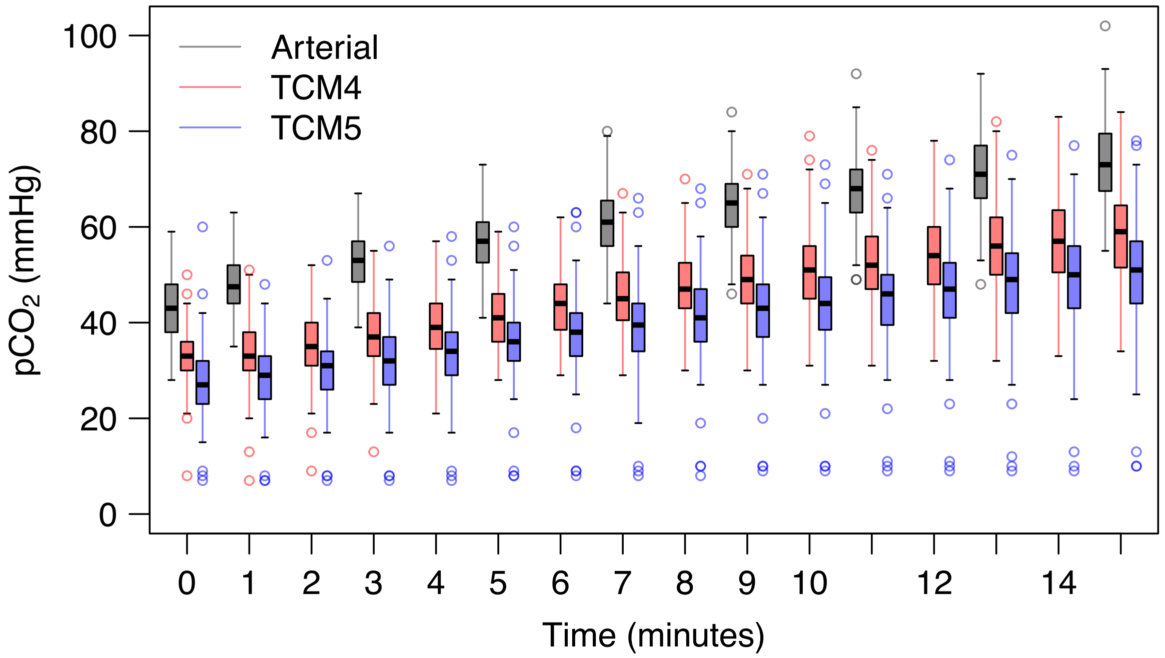

No. of patients:

Arterial	100	100	0	99	0	100	0	99	0	99	0	97	0	97	0	95
TCM4	91	91	91	91	91	91	91	92	91	91	90	89	89	89	88	87
TCM5	98	98	98	98	98	98	98	98	97	97	96	95	95	95	94	93

Figure S5. Box plots of CO_2 measurements at each time point using the two transcutaneous methods (TCM4 and TCM5) and arterial blood gas analysis based on the unfiltered data. Boxes indicate lower to upper quartiles and whiskers show the most extreme point within 1.5 times the interquartile range from the upper and lower quartile, respectively. Points beyond that range are indicated with circles.
