# Supplementary material for: Limitations of transcutaneous carbon dioxide monitoring in apneic oxygenation
Source: PLoS One. 2023 Jun 1;18(6):e0286038. doi: 10.1371/journal.pone.0286038 (PMC10234524; doi:10.1371/journal.pone.0286038)
Supplement: S1 Protocol — (DOCX) [file pone.0286038.s003.docx]

***PH*ysiology in *A*naesthesia *R*egarding *A*pnoeic *O*xygenation during nasal cannula therapy at different flow rates**

**Comparing high flow, medium flow and low flow nasal cannula therapy**

**A single-centre prospective randomized controlled trial.**

Clinical Study Protocol

Amendment 2

SHORT TITLE: PHARAO

| Study Type: | Other clinical trial |
| --- | --- |
| Study Categorisation: | Risk category A |
| Study Registration: | Clinical trials.org |
| Study Identifier: | PHARAO2018 |
| Sponsor-Investigator Principal Investigator: | Robert Greif  Lorenz Theiler  lorenz.theiler@insel.ch |
| Investigational Product: | not applicable, comparison of oxygenation methods |
| Protocol Version and Date: | V 4.0, 24.07.2019 |

The data underlying this study are ethically restricted per the Swiss Human Research Act, therefore data sharing is only possible with approval (https://www. swissethics.ch/). The name of the ethics committee is the Cantonal Ethics Committee Bern, Murtenstrasse 31, Hoersaaltrakt Pathologie, Eingang 43A, Buero H372, 3010 Bern, info.kek@be. ch (https://www.gsi.be.ch/de/start/ueber-uns/ kommissionen-gsi/ethikkommission.html). The data set name is "PHARAO_CO2." Interested researchers may contact the Direction of Division of Research, Department of Anaesthesiology and Pain Medicine, Bern University Hospital, Bern, Switzerland, anaesthesie@insel.ch (http://www. anaesthesiologie.insel.ch/de/; phone: +41 31 632 39 65).

Signature Page(s)

| Study number | Study registry and registration number (at clinicaltrials.gov) |
| --- | --- |
| Study Title | Physiology in anaesthesia regarding apnoeic oxygenation during nasal cannula therapy at different flow rates (PHARAO) |

The Sponsor-Investigator and trial statistician have approved the protocol version3.0,10.08.2018, and confirm hereby to conduct the study according to the protocol, current version of the World Medical Association Declaration of Helsinki, ICH-GCP guidelines or ISO 14155 norm if applicable and the local legally applicable requirements.

Sponsor: Prof. Dr. med. Robert Greif

Bern, 24.07.2019

| Place/Date |  | Signature |
| --- | --- | --- |

Principal Investigator

I have read and understood this trial protocol and agree to conduct the trial as set out in this study protocol, the current version of the World Medical Association Declaration of Helsinki, ICH-GCP guidelines or ISO 14155 norm and the local legally applicable requirements.

| Principal investigator: Prof. Dr. med. Lorenz Theiler |
| --- |

Bern, 24.07.2019

| Place/Date |  | Signature |
| --- | --- | --- |

Study Investigator: PD Dr. med. Heiko Kaiser

Bern, 24.07.2019

| Place/Date |  | Signature |
| --- | --- | --- |

Study Investigator: PD Dr. med. Thomas Riedel

Bern, 24.07.2019

| Place/Date |  | Signature |
| --- | --- | --- |

*Table of Contents*

Study synopsis 5

study summary in local language 10

1. STUDY ADMINISTRATIVE STRUCTURE 13

1.1 Sponsor-Investigator 13

1.2 Principal Investigator(s) 13

1.3 Statistician ("Biostatistician") 13

2. ETHICAL AND REGULATORY ASPECTS 13

2.1 Ethical considerations 13

2.1.1 The potential gain of new knowledge obtained with this study, and its meaning for patients/society: 13

2.1.2 Assessment of the benefit/risk relationship for the patient. 14

2.1.3 The methodology is ethically appropriate to gain new generalizable knowledge 14

2.2 Study registration 14

2.3 Categorisation of study 14

2.4 Competent Ethics Committee (CEC) 15

2.5 Ethical Conduct of the Study 15

2.6 Declaration of interest 15

2.7 Patient Information and Informed Consent 15

2.8 Participant privacy and confidentiality 15

2.9 Early termination of the study 16

2.10 Protocol amendments 16

3. Background and Rationale 16

3.1 Background and Rationale 16

3.2 Investigational Product (treatment, device) and Indication 17

3.3 Clinical Evidence to Date 17

3.4 Dose Rationale: Rationale for the intended purpose in study 17

3.5 Explanation for choice of comparator (or placebo) 18

3.6 Risks / Benefits 18

3.7 Justification of choice of study population 18

4. STUDY OBJECTIVES 19

4.1 Overall Objective 19

4.2 Primary Objective 19

4.3 Secondary Objectives 19

4.4 Safety Objectives 19

5. STUDY OUTCOMES 19

5.1 Primary Outcome 19

5.2 Secondary Outcomes 20

5.3 Safety Outcomes 20

6. STUDY DESIGN 20

6.1 General study design and justification of design 20

6.2 Methods of minimising bias 22

6.2.1 Randomisation 22

6.2.2 Blinding procedures 22

6.3 Unblinding Procedures (Code break) 22

7. STUDY POPULATION 22

7.1 Eligibility criteria 22

7.2 Recruitment and screening 23

7.3 Assignment to study groups 23

7.4 Criteria for withdrawal / discontinuation of participants 23

8. STUDY INTERVENTION 23

8.1 Identity of Investigational treatment 23

8.1.1 Experimental Intervention 23

8.1.2 Control Intervention (routine treatment) 23

8.2 Administration of experimental and control interventions 24

8.2.1 Experimental Intervention 24

8.2.2 Control Intervention 24

8.3 Data Collection and Follow-up for withdrawn participants 24

8.4 Study flow chart(s) / table of study procedures and assessments 25

8.5 Assessments of outcomes 26

8.5.1 Assessment of primary outcome 26

8.5.2 Assessment of secondary outcomes 26

8.5.3 Assessment of safety outcomes 26

8.6 Procedures at each visit 28

9. SAFETY 28

10. STATISTICAL METHODS 28

10.1 Hypothesis 28

10.2 Determination of Sample Size 28

10.3 Planned Analyses 29

10.3.1 Datasets to be analysed, analysis populations 29

10.3.2 Safety analysis 29

10.3.3 Deviation(s) from the original statistical plan 29

10.4 Handling of missing data and drop-outs 29

11. QUALITY ASSURANCE AND CONTROL 29

11.1 Data handling and record keeping / archiving 29

11.1.1 Case Report Forms 29

11.1.2 Record keeping / archiving 29

11.2 Data management 30

11.2.1 Data Management System 30

11.2.2 Data security, access and back-up 30

11.2.3 Analysis and archiving 30

11.2.4 Electronic and central data validation 30

11.3 Monitoring 30

12. PUBLICATION AND DISSEMINATION POLICY 31

13. FUNDING 31

13.1 Funding 31

14. INSURANCE 31

15. REFERENCES 31

Study synopsis

| Sponsor-Investigator | Prof. Dr. Robert Greif |
| --- | --- |
| Study Title: | Physiology in anaesthesia regarding apnoeic oxygenation during nasal cannula therapy at different flow rates |
| Short Title / Study ID: | PHARAO |
| Protocol Version and Date: | Version 4.0, 24.07.2019 |
| Trial registration: | Anticipated registry: [www.clinicaltrials.gov](http://www.clinicaltrials.gov) |
| Study category and Rationale | Other clinical trials. Risk category A.  Interventional RCT comparing four oxygenation methods to improve apnoea time |
| Clinical Phase: | not applicable |
| Background and Rationale: | After induction of general anaesthesia apnoea sets in. To ensure ventilation a tracheal intubation is then performed. If no measures to ensure ventilation are taken, desaturation will set in within minutes. To prolong the time to onset of desaturation pre-oxygenation is applied beforehand. Also, preparations to ensure swift tracheal intubation are made. Nevertheless, there is a phase, in which the patient is apnoeic, and deoxygenation may occur.  However, if anaesthesia needs to be provided in emergency situations or in the presence of a difficult airway, the rate of complication increases rapidly [1]. In these circumstances methods that reduce desaturation incidents during prolonged difficult intubation are desirable.  High-Flow nasal cannula therapy (HFNCT) is the administration of heated, humidified air/oxygen via nasal cannulas at rates of 50-100l/min. HFNCT was developed in intensive care unit as an alternative to continuous pressure support (CPAP). A new application for HFNCT in adults is the extension of apnoea time in patients with difficult airways who are undergoing general anaesthesia [2]. In this environment, a new term for this kind of oxygen therapy was created: THRIVE - transnasal humidified rapid-insufflation ventilatory exchange. Using HFNCT with continuous laryngoscopy, Patel et al. [2] demonstrated in fact a ventilatory effect with HFNCT.  THRIVE is thought to have a ventilatory effect, because slower than expected increase in pCO_2_ has been described [2, 3]. In these studies, researches compared their data to studies from the 1950-ies, where CO_2_ increase during apnoea was investigated [4, 5]. However, technology as well as patient factors have changed since then, and no study has ever directly compared different flow rates and the effect on CO_2_ clearance. Our previous research showed, that the claimed ventilatory effect is absent in children (Riva et al., British Journal of Anaesthesia, 2018, in press). Therefore, we want to systematically examine the CO_2_ clearance and possible influences in adults, and to determine, if a ventilatory effect is present at all.  This study investigates under controlled circumstances the concept of THRIVE to prolong the apnoea time without deoxygenation and to improve safety of airway management in patients. Furthermore, this study enables to quantify the physiologic effects of different flow rates of oxygen on carbon dioxide elimination in apnoeic patients as well as the effect of elevated CO_2_ levels on cerebral perfusion and other vital parameters.  During the study we observed that there is at best a minimal ventilatory effect in all groups regardless of flow. This could be because even at low flows, there are still turbulences occurring due to the excessive oxygen applied (for example, at 2l/min, 1.75l/min is excessive flow). It is already known that there is absolutely no ventilation (CO_2_ levels rise quicker) in obstructed airways (clamped tracheal tubes). It is unknown if there is a ventilatory effect in open airways without excessive flows. Therefore, in amendment 2, we add a fifth group where the oxygen applies equals to the oxygen absorbed in the body (about 0.25l/min). These 25 patients are being randomized within the existing randomization and continuously added for the reminder of the study. We intubate the fifth group of patients at the beginning of the study too ensure that all of the physiologically necessary 0.25l/min of oxygen are delivered to the patient.  Since we started the study, congress abstracts have postulated a flattening of the CO_2_ curve after about 20 minutes. To evaluate this theoretically possible flattening, we are expanding the apnoea time to a maximum of 30 minutes in a subset of 25 patients (5 in each group). In their landmark publication of 2013, Patel and Nouraei reported apnoea time of up to 65 minutes without problematic side effects to the patients. The respiratory acidosis is fully reversible within minutes, which will be demonstrated by an additional blood gas measurement 15 minutes after termination of the study intervention. |
| Objective(s): | This study compares under controlled conditions if different flow rates and physiologic apnoeic oxygenation affect apnoea time after induction of anaesthesia and how CO_2_ clearance is influenced. Furthermore, this study enables to quantify the effects of increased pCO_2_ on cerebral perfusion.  Our primary hypothesis states that any of the three intervention is non-inferior to HFNCT regarding clearance of CO_2_ (ventilatory effect). |
| Outcome(s): | Primary outcome: mean p_tc_CO_2_ increase in mmHg/min during apnoea time, measured transcutaneously.  Secondary outcomes: in case of desaturation within the predefined apnoea time: time (in seconds) until desaturation from SpO_2_ 100% to 92%, measured by pulse oximetry. Changes in p_tc_O_2_, p_a_O_2_ and p_a_CO_2_ in mmHg/min, blood gas analysis parameters, upper airway patency, influence of jaw thrust vs. continuous laryngoscopy on CO_2_ clearance during HFNCT, changes in brain oxygenation (measured by near infrared spectroscopy (NIRS)) during apnoea time, changes in electrical impedance tomography (EIT) to validate ventilatory effect and pulse contour analysis to monitor cardiac output. |
| Study design: | 1. Prospective, single centre, randomised controlled trial with five groups: low flow 2 l/min using jaw thrust 2. medium flow 10 l/min using jaw thrust 3. high flow 70 l/min using continuous laryngoscopy 4. high flow 70 l/min of oxygen, using jaw thrust 5. minimal flow 0.25l/min of oxygen via an endotracheal tube |
| Inclusion / Exclusion criteria: | We will include adult patients undergoing general anaesthesia for elective surgery at the Dept. of Anaesthesiology and Pain Therapy, Bern University Hospital, Inselspital, Bern, ASA physical health status 1-3, who have provided written informed consent in German or French.  Exclusion criteria are:   - Any Indication for fibre optic intubation - Expected impossible mask ventilation - Known coronary heart disease - Known heart failure, NYHA classification ≥ 2 - Arrhythmias in need of anti-arrhythmic therapy (e.g. implanted cardio defibrillator) - Peripheral occlusive arterial disease, Fontaine ≥ 2b - Treatment with beta-receptor antagonists - Known stenosis of the (common or internal) carotid or vertebral arteries - BMI > 35kg/m^2^ and BMI < 16kg/m^2^ - Hyperkalaemia (K > 5.5 mmol/l) - Known COPD Gold classification ≥ 2 - Known pulmonary arterial hypertension, systolic > 35mmHg - Known obstructive sleep apnoea syndrome in need of therapy - High risk of aspiration (requiring rapid sequence induction intubation) - Increased intracranial pressure - Intracranial surgery - Limited knowledge of German or French language - Absent power of judgement - Anaemia, Hb < 100 g/l - Pregnancy (pregnancy test in all female patients prior to anaesthesia) - Neuromuscular disorder - Known or suspected cervical spine instability - Nasal obstruction, impossibility of nasal ventilation (both sides patent) - Allergies or contra-indications to one or more of the used anaesthesia agents - Age > 80 years   Randomization will be stratified according to BMI (16-25kg/m^2^, 25-30 kg/m^2^, 30-35 kg/m^2^), and smoker status (non-smoker, , daily smoker < 40 years of age, daily smoker > 40 years of age, former smoker). |
| Measurements and procedures: | Eligible, consenting adults will be prepared for general anaesthesia in the usual way consisting of EKG, pulse-oximetry, Narcotrend^TM^, a venous cannula and an arterial line for continuous blood pressure monitoring. They will receive additional non-invasive monitoring for this study, such as transcutaneous measurement of pCO_2_ and O_2_, NIRS, and thoracic electrical impedance tomography (EIT, PulmoVista^®^ 500, Draeger, Luebeck, Germany)  Standard pre-oxygenation (until etO_2_ is > 90% or time > 3 minutes) will be done. Anaesthesia will be started (= ”induction”) using Propofol and Fentanyl i.v., using Narcotrend^TM^ to measure depth of anaesthesia. All patients will receive a standard dose (Rocuronium, 0.9mg/kg) of neuromuscular blockage to facilitate airway management and total intravenous anaesthesia will be installed to maintain anaesthesia. Train of four measurement (TOF) will be confirmed full neuromuscular blockage with Rocuronium every 30 seconds. If necessary, additional dosage of neuromuscular blockage will be administered.  After administration of Rocuronium, possibility of mask ventilation will be confirmed and the sealed envelope with the randomization will then be opened. As a study intervention, the assigned method (HFNCT 70 l/min with either jaw thrust or laryngoscopy, or 10 l/min or 2l/min with the standard nasal canula, or 0.25l/min delivery of oxgen via a tracheal tube) will be installed and mask ventilation discontinued starting the apnoea period. Nasopharyngoscopy (EF-N slim, Acutronic, Hirzel, Switzerland) will confirm upper airway patency. Blood gas analysis will be conducted: baseline awake, start of apnoea, first minute after apnoea start, and every 2 minutes thereafter with a maximum of 150 ml in total. Other measurements (ECG, pulse-oximetry, blood pressure, NIRS, thoracic EIT, Narcotrend^TM^, P_tc_O_2_, P_tc_CO_2_) will be measured continuously over the study period.  The study intervention will end when one of the following criteria (study end-points) is met: SpO_2_ <92%, P_tc_CO_2_ > 100 mmHg or time > 30 minutes.  When any of the end points is reached, patient-centred standard anaesthesia care will be continued, as planned for the case.  A post-operative interview will be conducted on the first post-operative day to evaluate injuries during airway management (e.g. bleeding, sore throat, hoarseness, lip injuries), pain, postoperative nausea and vomiting. |
| Study Intervention: | Group 1: nasal cannula, 100% oxygen at 2 l/min (low flow)  Group 2: nasal cannula, 100% oxygen at 10 l/min (medium flow)  Group 3: HFNCT, 100% oxygen at 70 l/min, jaw thrust  Group 4: supply of 100% oxygen at 0.25l/min via an endotracheal tube |
| Control Intervention: | Group 5: HFNCT, 100% oxygen at 70 l/min, continuous laryngoscopy |
| Number of Participants with Rationale: | We include 25 patients per group as we chose a non-inferiority design for our study. If the difference of means is less than 0.3 mmHg/min, we assumed clinical equivalence: Even if HFNCT was used as THRIVE for 30 minutes, this difference would mount to 9mmHg, less than the normal range of CO_2_ in arterial blood gas analysis [6] Assuming a standard deviation of 0.35 [3], an alpha of 0.025 and a power of 80%, this calculates to a sample size of 22. Accounting for drop-outs and missing data, we include 25 patients per group, or 100 patients overall. The statistical analysis concept does not need to be adjusted with the addition of a fifth group, we will still need 25 patients per group, because we are comparing each group individually to the control group. |
| Study Duration: | 2 years |
| Study Schedule: | March 2018 – March 2020 |
| Investigator(s): | Prof. Dr. med. Lorenz Theiler, Prof. Dr. med. Robert Greif, Dr. med. Heiko Kaiser, all at the Dept. Anaesthesia and Pain Therapy, and PD Dr. med. Thomas Riedel, Department of Paediatric Intensive Care Medicine, University Hospital, Inselspital, Bern  Lorenz Theiler, lorenz.theiler@insel.ch, phone 0316320804, Address Inselspital Freiburgstrasse, 3010 Bern |
| Study Centre: | Inselspital Bern |
| Statistical Considerations: | Distribution of data will be checked using qq-plots and swilk command in stata, normal distributed data will be presented as means and standard deviation, otherwise median and interquartile range will be used. Proportions will be presented as numbers and percentage. Analytical statistics uses Mann-Whitney u-tests and Students t-test, according distribution, or ANOVA and Kruskal-Wallis for multiple groups. Each of the groups will a-priory be compared to the control group independently, without correction of multiple comparisons. Other multiple comparisons or subgroup-analyses will be performed using correction factors. Proportions will be compared by Chi-square tests or Fisher’s exact test. As sensitivity analyses, we will do regression analyses, using mixed effects linear regressions. There are no adjustments to this because of the new additional group in amendment No. 2 |
| GCP Statement: | This study will be conducted in compliance with the protocol, the current version of the Declaration of Helsinki, the ICH-GCP or ISO EN 14155 (as far as applicable) as well as all national legal and regulatory requirements. |

study summary in local language

Apnoe-Oxygenierung geht in der Geschichte der Anästhesiologie weit zurück. Dies hat deshalb einen grossen Wert, weil es bei jeder Allgemeinanästhesie zu einer kurzen Phase der Apnoe kommt, bevor der tracheale Tubus platziert werden kann. Dieses Prozedere ist sehr sicher. Sehr selten kann es aber zu unerwarteten Schwierigkeiten kommen, die innerhalb weniger Minuten zu einer Entsättigung des Patienten führen. Möglichkeiten diese Zeit zu verlängern sind deshalb von Patientensicherheitsseite wünschenswert.

In den letzten Jahren ist insbesondere die Anwendung von HFNCT (high flow nasal cannula therapy, Therapie via Nasensonde mit hohen Flussraten) im Bereich der trachealen und laryngealen Chirurgie in den Vordergrund getreten. Hierfür wird Sauerstoff erwärmt und angefeuchtet, damit er unter Flussraten von bis zu 70 l/min über die Nase insuffliert werden kann. Unter dem Namen THRIVE (transnasal humidified rapid-insufflation ventilatory exchange) wurde dieses Verfahren bei Patienten mit kontinuierlicher Laryngoskopie etabliert. Dieser Name ist durch lange Apnoezeiten (bis 65 Minuten), und durch den beobachteten, langsamer als erwarteten CO_2_ Anstieg gekennzeichnet. Es wird angenommen, dass durch die hohen Flussraten Turbulenzen entstehen, die sich über die Trachea bis in die Alveolen fortsetzen und somit zu einer gewissen CO_2_-Auswaschung führen, was aber bisher nicht bewiesen werden konnte.
Eine kürzlich durch unsere Studiengruppe durchgeführte Studie von Riva et al. (British Journal of Anaesthesia, 2018, in press) an Kindern widerlegte allerdings diesen Ventilations-Effekt bei Kindern. Die Datenlage bei Erwachsenen ist noch lückenhaft, insbesondere bei unterschiedlichen Flussraten und deshalb wollen wir hier eine genauere Untersuchung starten.

Dafür sollen insgesamt 100 Patienten rekrutiert werden, die im Rahmen eines elektiven chirurgischen Eingriffs eine Allgemeinanästhesie erhalten müssen. Unsere Untersuchung wird dafür in den normalen Ablauf der Anästhesie-Einleitung eingebettet.
Bevor die Anästhesie eingeleitet werden kann, muss der Patient präoxygeniert werden. Anschliessend wird die Narkose eingeleitet. Wenn der Patient bewusstlos ist, wird mit Maskenatmung begonnen und die randomisierte Zuteilung in die unterschiedlichen Interventionsgruppen aufgedeckt.
Eine der folgenden fünf Massnahmen wird anschliessend durchgeführt: THRIVE mit 70 l/min unter Anwendung des Esmarch Manövers (jaw thrust), THRIVE mit 70 l/min unter permanenter Laryngoskopie (Kontroll-Gruppe), 10 l/min mit Esmarch, 2 l/min mit Esmarch oder 0,25l/min Sauerstoff direkt über den Beatmungsschlauch.

Währenddessen werden EKG, Pulsoxymetrie, Narcotrend^TM^, transkutanes pCO_2_ und pO_2_, NIRS, Elektrische-Impedanz-Tomographie, Blutdruck und Herzzeitvolumen (mit FloTrac über Pulsdruckkurven) kontinuierlich aufgezeichnet. Ausserdem werden alle zwei Minuten arterielle Blutgasanalysen durchgeführt.
Die zugeteilte Massnahme erfolgt bis entweder S_a_O_2_ < 92%, P_tc_CO_2_ > 100mmHg oder Zeit > 30 Minuten aufgetreten sind.
Anschliessend wird der Atemweg gesichert und normal mit der Anästhesie fortgefahren. Bevor der Patient entlassen wird, führen wir eine Befragung durch, wo allfällige Nebenwirkungen und Komplikationen des Atemwegmanagements erfragt werden.
Unsere Hauptfrage ist, wie unterschiedliche Flussraten und Mundöffnung die CO_2_ Auswaschung beeinflussen, wobei wir davon ausgehen, dass die tieferen Flussraten nicht relevant schlechter sind. Zusätzlich werden wir mit NIRS nicht invasiver Messung über der Haut der Stirn den Effekt des erhöhten CO_2_ auf die zerebrale Durchblutung messen, mittels EIT bestimmen wir Änderungen der funktionellen Residualkapazität der Lungen und wir können über Veränderungen Aussagen über potentiell auftretende Atelektasen treffen. Ausserdem können auch arterielle Messungen von pO_2_ und pCO_2_ mit den transkutanen Messungen verglichen werden.

Durch die Erkenntnisse dieser Studie erwarten wir, in Zukunft die apnoeische Oxgenation auch mit niedrigeren Flussraten durchführen zu können, was nicht nur weniger O_2_ verbraucht, sondern auch materialtechnisch gesehen deutlich billiger ist. Auch ist die Verfügbarkeit von normalen Nasenbrillen in jedem Operationssaal und sogar auf Abteilungen überall gegeben, wohingegen THRIVE-basierte Systeme deutlich seltener vorhanden sind. Insbesondere bei einem Atemwegsnotfall wäre eine schnell verfügbare Alternative wichtig, um eine schnelle Entsättigung bei prolongierter Atemwegsicherung zu vermeiden. Schliesslich gibt es keine Evidenz, welche physiologische Veränderungen bei anästhesierten Patienten stattfinden, die nun routinemässig bis zu einer Stunde ohne Ventilation oxygeniert werden.

**Abbreviations**

| AE | Adverse Event |
| --- | --- |
| BMI | Body-mass index |
| BP | Blood pressure |
| CA | Competent Authority (e.g. Swissmedic) |
| CEC | Competent Ethics Committee |
| CPAP | Continuous positive airway pressure |
| CO_2_ | Carbon Dioxide |
| CRF | Case Report Form |
| EIT | Electrical Impedance Tomography |
| GCP | Good Clinical Practice |
| Ho | Null hypothesis |
| H1 | Alternative hypothesis |
| HFG  HFNCT | Humanforschungsgesetz (Law on human research)  High Frequency Nasal Cannula Therapy |
| HMG | Heilmittelgesetz |
| HRA | Federal Act on Research involving Human Beings |
| IIT | Investigator-initiated Trial |
| ITT | Intention to treat |
| KlinV | Verordnung über klinische Versuche in der Humanforschung *(in English: ClinO)* |
| l/min | Liters per minute |
| MD | Medical Device |
| NIRS | Near infra-red spectroscopy |
| p_a_ | Arterial partial pressure |
| p_tc_ | Transcutaneous partial pressure |
| O_2_ | Oxygen |
| PI | Principal Investigator |
| SDV | Source Data Verification |
| SOP  THRIVE | Standard Operating Procedure  transnasal humidified rapid-insufflation ventilation exchange |
| TOF | Train of four |

**Study schedule**

March 2018 Start clinical part of the study, first patient included

March 2020 end of study

# STUDY ADMINISTRATIVE STRUCTURE

Prof. Dr. med. R. Greif, Prof. Dr. med. L. Theiler, and PD Dr. med. H. Kaiser are members of the Department of Anaesthesia and Pain Therapy at the Bern University Hospital Inselspital, Freiburgerstrasse, 3010 Bern. PD Dr. med. T. Riedel is a member of the Department of Paediatric Intensive Care Medicine, University Hospital, Inselspital, Bern

Contact details for the PI Lorenz Theiler: Lorenz Theiler, Prof. Dr. med. lorenz.theiler@insel.ch 031 632 0804

## Sponsor-Investigator

Prof Robert Greif will overview the entire study, the protocol drafting and final version, the analysis of the results and the writing of the manuscript for publication. Contact details: robert.greif@insel.ch, 0316321629

## Principal Investigator(s)

Lorenz Theiler, Prof. Dr. med. lorenz.theiler@insel.ch 031 632 0804

## Statistician ("Biostatistician")

Lorenz Theiler, Prof. Dr. med. lorenz.theiler@insel.ch 031 632 0804

# ETHICAL AND REGULATORY ASPECTS

Before the study will be conducted, the protocol, the proposed patient information and consent form as well as other study-specific documents will be submitted to the Cantonal Ethics Committee Bern. Any amendment to the protocol must as well be approved by this institution.

The decision of the CEC concerning the conduct of the study will be made in writing to the Sponsor-Investigator before commencement of this study. The clinical study can only begin once approval from all required authorities has been received. Any additional requirements imposed by the authorities shall be implemented.

## Ethical considerations

### The potential gain of new knowledge obtained with this study, and its meaning for patients/society:

With this study, we will be able to determine, if high flow nasal cannula therapy (HFNCT) provides a real benefit over medium flow or low nasal cannula therapy. This can be of use in emergency situations, where airway access is sometimes difficult to obtain as apnoeic oxygenation could prolong apnoea time until desaturation. Lower flow rates can be provided in almost all clinical settings, including preclinical situations and on medical wards through already available systems. For HFNCT, a specific device is necessary, the availability of which is very limited. If non-inferiority can be shown, it could result in a greater safety for all patients through widespread availability of oxygen therapy devices, at a reduced cost compared to HFNCT. Furthermore, there is no evidence available about the physiological changes in anaesthetised patients undergoing HFNCT for a prolonged time, which now has been established as a standard procedure.

### Assessment of the benefit/risk relationship for the patient.

HFNCT and apnoeic oxygenation are concepts that have been provided with enough evidence to sustain their acceptance in clinical use. There have even been studies suggesting possibility of oxygenation, using very low flows of oxygen [7-9]. But both, high flow and low flow have never been directly compared for its influence on human physiology.
However small the risk, desaturation in low-flow groups cannot be completely ruled out. But one of our endpoints is saturation of <92% after which the experiment will be discontinued. Saturation of 92% does not lead to any ischemic damage in healthy patients and all patients with known risks or vulnerability of ischemic events are excluded.
Furthermore, p_a_CO_2_ will rise, this leads to a cerebral vasodilation. There is no data setting any threshold after which potential damage would occur, even though patients with p_a_CO_2_ up to 250 mmHg have been shown to recover without any neurological impairment [4]. Our limit will be set at 100 mmHg, which therefore should not lead to any neurological damage whatsoever, especially since all patients with high vulnerability towards hypercapnia are excluded. The respiratory acidosis is fully reversible within minutes, which will be demonstrated by an additional blood gas measurement 15 minutes after termination of the apnoea time. Measuring near infrared spectroscopy (NIRS) will furthermore ensure, that cerebral perfusion will stay within normal limits.

CO_2_ is known to have haemodynamic influence [10, 11], but our pilot data suggest that anaesthetic drugs like Propofol and Remifentanil overrule this effect, as median blood pressure never exceeded 80mmHg.

Patients safety will not be compromised during the study time, as this procedure has shown to prevent desaturation up to 65 minutes [2, 12]. This fact combined with the experience that we made during the course of our various THRIVE-researches, makes us confident to look for the development of pCO_2_ in the five study groups if we prolong the measurement time to 30 minutes. If unexpected difficult airway should occur in these patients, there is more time for securing the airway until desaturation will set in.

### The methodology is ethically appropriate to gain new generalizable knowledge

The patients are blinded to the randomisation. The responsible anaesthetist cannot influence the apnoea time (very low risk of creating bias). The five groups will be stratified according to BMI (16-25kg/m^2^, 25-30 kg/m^2^, 30-35 kg/m^2^), smoker status (non-smoker, daily smoker, former smoker) and age (18-40 years, > 40 years) in order to maintain statistical equality. The trial method is well suited to study the research question and the participating anaesthetists are comfortable and used to all four oxygenation possibilities. All patients that could be harmed through high levels of CO_2_ or desaturation are excluded from the study. The study group has a wide experience with HFNCT and has performed a similar study in children before (Riva et al, BJA, 2018, in press).

## Study registration

The study will be registered at www.clinical-trials.org, and at the database of the Inselspital Bern through the Clinical Trial Unit of the University of Bern, as well as the KOFAM of the BAG (www.kofam.ch).

## Categorisation of study

Risk category A.

Rationale: No medication or device will be investigated. We compare a therapeutic strategy in three experimental and one standard therapy arm (= oxygen application is used every day in anaesthesia in this setting).

## Competent Ethics Committee (CEC)

Kantonale Ethikkommission Bern (KEK), Institut für Pathophysiologie, Hörsaaltrakt Pathologie, Eingang 43A, Büro H372, Murtenstrasse 31, 3010 Bern

## Ethical Conduct of the Study

The study will be carried out in accordance to the protocol and with principles enunciated in the current version of the Declaration of Helsinki, the guidelines of Good Clinical Practice (GCP) issued by ICH, in case of medical device: the European Directive on medical devices 93/42/EEC and the ISO Norm 14155 and ISO 14971, the Swiss Law and Swiss regulatory authority’s requirements. The CEC and regulatory authorities will receive annual safety and interim reports and be informed about study stop/end in agreement with local requirements. A final report will be sent at least one year after study stop/end to CEC.

## Declaration of interest

There is no conflict of interest by any of the investigators.

## Patient Information and Informed Consent

The investigator will explain to each participant the nature of the study, its purpose, the procedures involved, the expected duration, the potential risks and benefits and any discomfort it may entail. Each participant will be informed that the participation in the study is voluntary and that he/she may withdraw from the study at any time and that withdrawal of consent will not affect his/her subsequent medical assistance and treatment.

The participants must be informed that the medical records may be examined by authorised individuals (e.g. CEC) other than their treating physician.

All participants of the study will be provided a study information sheet with a consent form describing the study and providing sufficient information for the participant to make an informed decision about their participation in the study at their anaesthesia visit before surgery.

The study information sheet with the consent form will be submitted to the CEC to be reviewed and approved. The formal consent of the participant, using the approved consent form, must be obtained before the participant is submitted to any study procedure.

The study participant should read and consider the statement before signing and dating the informed consent form and should be given a copy of the signed document. The consent form must also be signed and dated by the investigator (or his designee) and it will be retained as part of the study records.

## Participant privacy and confidentiality

The investigators affirm and uphold the principle of the participant's right to privacy and that they shall comply with applicable privacy laws. Especially, anonymity of the participants shall be guaranteed when presenting the data at scientific meetings or publishing them in scientific journals.

Subject confidentiality will be ensured by utilising subject identification code numbers to correspond to treatment data in the computer files. The patient code is build using the patient identifier (from the Inselspital’s SAP) multiplied by two. The patient identifier can only be obtained by Inselspital personnel.

The code will be stored in a lockable cupboard in the lockable office of the principal investigator where no one else has access.

For data verification purposes, authorised representatives of a competent authority (e.g. Swissmedic), or the cantonal ethics committee may require direct access to parts of the medical records relevant to the study, including participants’ medical history.

## Early termination of the study

The Sponsor-Investigator may terminate the study prematurely according to certain circumstances, for example:

- ethical concerns
- insufficient participant recruitment
- when the safety of the participants is doubtful or at risk, respectively
- alterations in accepted clinical practice that make the continuation of a clinical trial unwise
- early evidence of harm of the experimental intervention

## Protocol amendments

Substantial amendments are only implemented after approval of the CEC. Under emergency circumstances, deviations from the protocol to protect the rights, safety and well-being of the study participants may proceed without prior approval of the sponsor and the CEC. Such deviations shall be documented and reported to the sponsor as soon as possible.

All Non-substantial amendments are communicated to the CEC within the Annual Safety Report (ASR).

# Background and Rationale

## Background and Rationale

Normally, after induction of general anaesthesia apnoea sets in. While waiting for optimal intubation conditions, manual mask ventilation ensures ventilation and oxygenation. An intubation is performed so the airway is secured, and ventilation can be operated mechanically by a respirator. If no measures to ensure ventilation after anaesthesia induction were taken, desaturation would set in within minutes.

Pre-oxygenation is part of preparation initiated before induction in order to fill up oxygen reserves in the patient’s body (e.g. lungs and haemoglobin). Also, preparations to ensure swift tracheal intubation are made (e.g. neuromuscular blockage). Nevertheless, there is a phase, in which the patient is apnoeic, and a deoxygenation may occur. This phase, however short, cannot be avoided while placing the tube into the trachea.

If anaesthesia needs to be provided in emergency situations or in presence of a difficult airway, the rate of complication increases [1]. This is due to hindered or impossible preparation, difficult circumstances and prolonged intubation and therefore apnoea-time. In these circumstances methods to reduce desaturation incidents are desirable.

High-Flow nasal cannula therapy is the administration of heated, humidified oxygen via nasal cannula at rates up to 100 l/min. This high-flow nasal cannula therapy was developed in the intensive care unit as an alternative to continuous pressure support (CPAP).

A new application for high-flow nasal cannula therapy in adults is the extension of apnoea time in patients with difficult airways who are undergoing general anaesthesia [2]. In this environment, a new term for this kind of oxygen therapy was created: THRIVE - transnasal humidified rapid-insufflation ventilatory exchange. It was described to have prolonged apnoea time up to 65 minutes [2, 3, 13].

THRIVE has shown to be a suitable method to prolong apnoea with stable O_2_ saturation [2, 3]. Despite promising pilot results with high-flow nasal cannula therapy, it is unknown whether this technique is superior to low-flow nasal cannula therapy. Especially the claimed ventilatory effect has not yet been provided with sufficient data. It is thought to happen through turbulences caused by the high flow, that continue down through the trachea into the alveoli. This turbulence is said to washed out CO_2_ because slower increase of CO_2_ has been described using THRIVE vs. normal apnoea. However, this data of normal apnoea was already acquired in 1959 by Frumin et al. on only 8 healthy patients [4]. Since then, many things, including technology and patient factors have changed and this data therefore has only limited application today.

Especially the CO_2_ clearance under different flow rates has yet to be thoroughly examined. This is important, as increased CO_2_ can cause a multitude of effects like acidosis and altered cerebral blood flow [4, 11, 14]. Our study will also examine the effect of increasing p_a_CO_2_ on cerebral perfusion using NIRS. To judge this effect in its entirety, we have to examine the development of the CO_2_-curve for a longer period of time to differentiate whether the increase of CO_2_ is constant, logarithmic or of any other significance. We want to verify the postulated pathophysiological mechanism of CO_2_ buffers over time. We hypothesize that such a time course does not exist and other mechanism are responsible for the constant increase in CO_2_.

Furthermore, we will compare HFNCT at 70 l/min performing jaw thrust, with a group of patients, whose mouths will be opened using continuous video laryngoscopy, which is a standard procedure today for laryngeal surgery. In our study, we will only apply the minimal strength required in order to keep the airway patent, which can be visually confirmed using the video-monitor. Whether laryngoscopy compared to jaw thrust makes any difference in oxygenation or CO_2_ increase has yet to be examined.

This study investigates under controlled circumstances the concept of THRIVE to prolong apnoea time without desaturation. With our findings, we hope to improve airway management safety. Furthermore, this study enables us to better understand physiology behind apnoeic oxygenation and the influence of different nasal flow rates. Our study’s extensive setup also allows us to measure the effects of increased CO_2_ on cerebral blood flow and cardiac output.

## Investigational Product (treatment, device) and Indication

Not applicable, since we are evaluating a concept, which is already in routine use, not a device.

## Clinical Evidence to Date

All relevant available evidence is summarized under 3.1

## Dose Rationale: Rationale for the intended purpose in study

High Flow (70 l/min): THRIVE is normally used with very high flows, this is standard procedure
Medium Flow (10 l/min): Every anaesthetic respirator or oxygen wall connection can provide 10 l/min, which in emergency situation would be superior due to its higher availability.
Low Flow (2 l/min): Low flows could even be used with dry oxygen. This would reduce cost even further, and could find application in prevention of desaturation in high risk situations. Minimum Flow (0.25 l/min): 250ml is approximately the amount of oxygen that is resorbed in the lungs each minute. No turbulences in the airway will occur due to the streaming oxygen.

## Explanation for choice of comparator (or placebo)

Our control group consists of standard of care use when using HFNCT. They will receive 70l/min using continuous laryngoscopy as it has been established by Patel et al. [2]. We will not use a placebo (which could be air (21% oxygen)) for this study, as this would lead to desaturation within a few minutes and is not standard of care.

However, HFNCT is not standard of care during anaesthesia induction as patients normally receive mask-preoxygenation, mask ventilation and tracheal intubation while the apnoea period is kept as short as possible.

## Risks / Benefits

Low-flow oxygen is being used every day to virtually every patient on medical wards, intensive care units or postoperative care units, and in the operating theatre. High-flow oxygen is being used in adult anaesthesia practice for a few years now with oxygen flow rates of 0.5-70 l/min in the operating theatre during anaesthesia, and have very little complications.

Drying of the nasal mucosa can cause discomfort in patients with prolonged nasal cannula therapy, especially if the oxygen is not humidified [15]. We will provide pre-nasal cannulas which have been shown to be less likely to cause discomfort [16]. Additionally, we will only use humidified oxygen. Patients receiving high flow will also profit from additionally heated oxygen delivery, which even further reduces nasal irritations.

Further risk is due to placement of an arterial catheter (e.g. obstruction of vessel or bleeding). Risks are overall extremely low [17]. Risk factors for complications include peripheral occlusive arterial disease, but we exclude these patients from our study, further reducing risk of complications. Also, an experienced anaesthetist will place this cannula to minimize risk for multiple attempts.
For an arterial blood gas analysis, very little blood is needed, and we estimate to take a maximum of 150 ml of blood in total from each patient. As we have excluded all patients with haemoglobin < 100 g/l, this amount can be considered haemodynamically irrelevant and will not trigger transfusion during surgery.

A very small risk for mucosal bleeding occurs, when passing the nasopharyngoscope through the nose, to obtain view of the larynx. In normal preoperative airway assessment this procedure is regularly performed on awake patients and we have never witnessed a mucosal bleed. Nevertheless, this will be further reduced, by having a properly trained and well experienced operator performing this task.

EIT, NIRS, Narcotrend^TM^, heart rate, and transcutaneous capnography are all very standard, non-invasive measurements that can be obtained in an extremely safe manner.

## Justification of choice of study population

The study participants are of all ages and genders. We want a heterogenic population presenting to the operating theatre each day. We exclude patients who are too sick to safely undergo our experiment or who could bias our physiological findings with their underlying disease.

For more see 7.1: eligibility criteria.

# STUDY OBJECTIVES

## Overall Objective

This study investigates under controlled conditions the concept of nasal cannula therapy at different flow rates to prolong the apnoea time during induction of anaesthesia. We hope to provide evidence that can further improve the safety of airway management in adult patients. This study will enable us to quantify the physiological effects of high-flow nasal cannula therapy and help us to distinguish it better from conventional medium and low-flow apnoeic oxygenation. Especially differences in p_tc_CO_2_ will be observed and compared in order to determine the effect of nasal flow on CO_2_ clearance as well as the effects of elevated CO_2_ levels on vital parameters.

## Primary Objective

Primary study objective is to evaluate the mean increase of transcutaneous CO_2_ (p_tc_CO_2_) in mmHg/min.

## Secondary Objectives

Secondary study objective is to evaluate the mean duration of apnoea. Also, changes in EIT, NIRS, BP and heart rate are measured. We will also assess airway patency while performing jaw thrust. This can be either patent or not-patent.
We also want to monitor the effect of increased CO_2_ on the cardiac output via the simultaneously obtained pulse contour analysis using either FlowTrac by Edwards Lifescience, Irvine, California, or LiDCO by LiDCO Ltd, London, UK.

Bilateral frontal cerebral NIRS measurements will be performed to determine the effect of high CO_2_ on brain perfusion. A NIRO monitor by Hamamatsu, Japan, will be used, which delivers brain oxygenation as a surrogate of brain perfusion, as well as the percentage of oxygenated and deoxygenated blood in the sampled tissue.

To ensure absence of respiratory movements, complete neuromuscular blockage will be confirmed using train-of-four (TOF) which should be 0 throughout the experiment. Additional muscle relaxants may be administered if necessary. Respiration must be avoided at all times, since otherwise our results would be unusable.

## Safety Objectives

Saturation using simple pulse-oximetry will be closely monitored and any desaturation below 92% will lead to immediate discontinuation of the experiment. NIRS will be measured to register all changes in cerebral blood flow that might be triggered by increased p_a_CO_2_.

# STUDY OUTCOMES

## Primary Outcome

Primary outcome: increase of p_tc_CO_2_ level in mmHg/min during apnoea.

## Secondary Outcomes

Secondary outcomes:

- Seconds until desaturation from SpO2 100% to 92% measured by pulse oximetry (if occuring at all)
- Lowest saturation
- p_tc_O_2_ rise in mmHg/min
- Airway patency (yes/no)
- Vital parameters (BP, HR, SpO_2_)
- Arterial pH, p_a_CO_2_ and p_a_O_2_
- Cerebral Oxygenation (NIRS)
- Highest etCO_2_ measured in the first minute after securing airway in mmHg
- TOF
- Cardiac output
- Changes in lung volume measured by EIT

## Safety Outcomes

The patient is monitored during the whole procedure to detect hypoxemia (SpO2 <92%) or hypercapnia (p_tc_CO_2_ > 100 mmHg).

# STUDY DESIGN

## General study design and justification of design

Single blinded randomised controlled prospective trial (High-flow 70 l/min using two different techniques, medium-flow 10 l/min and low-flow 2 l/min, each with 100% oxygen).

Four interventions will be applied.

- High-flow nasal cannula oxygen **70** l/min using OptiFlow™ system by Fisher&Paykel™ and an oxygen inspiration concentration F_i_O_2_ of 100%, with continuous laryngoscopy
- High-flow nasal cannula oxygen **70** l/min using OptiFlow™ system by Fisher&Paykel™ and an oxygen inspiration concentration F_i_O_2_ of 100%, performing jaw thrust
- Medium-flow nasal cannula oxygen **10** l/min using Carbamed digiflow, Aquapak Hudson RCI to humidify oxygen and standard nasal cannula with 100% oxygen, performing jaw thrust
- Low-flow nasal low-flow oxygen **2** l/min using Carbamed digiflow, Aquapak Hudson RCI and standard nasal cannula with 100% oxygen, performing jaw thrust
- Minimum-flow oxygen 0.25l/min with 100% oxygen, using standard tracheal tubes.

The departmental digital Anaesthesia Information System (AIS) will be used to screen patients for compatibility.

Once written informed consent is obtained, after patients were given enough time to validate their participation and considering all possibilities, and pregnancy has been excluded in female patients (in all female patients: pregnancy test before inclusion), the patient will be brought to the operating theatre. After local anaesthesia, an arterial cannula will be placed into the radial artery and arterial blood gases will be drawn. All necessary monitoring (EKG, pulse oximetry, EIT, NIRS, Narcotrend^TM^, transcutaneous pCO_2_) will be installed and pre-oxygenation will begin. Once etO_2_ has reached 90% or more than three minutes have passed, induction of anaesthesia will begin using a TCI system for both Propofol and Remifentanil. For neuromuscular blockage, Rocuronium 0.9mg/kg will be administered.

After induction of general anaesthesia confirmed by absent respiratory movement, unconsciousness, and according Narcotrend^TM^-values; mask ventilation will commence. If for whatever reason mask ventilation is still impossible after full relaxation and placement of a Guedel tube, the experiment will be discontinued immediately, and the airway will be secured by the anaesthetist.

According to randomization, the intervention will be applied. If jaw thrust is performed, upper airway patency will be visually confirmed using nasopharyngeal fiberscope (EF-N slim, Acutronic, Hirzel, Switzerland). In most cases this should be the case [18]. If not, an oropharyngeal airway (Guedel) will be inserted, and if necessary, manual assistance performed to obtain the required result. If, despite these interventions, the airway is not patent, the study will end prematurely (drop-out). This is very unlikely to happen.
After confirmation of upper airway patency, the experiment will be continued until one of the following criteria is met: SpO_2_ < 92%, p_tc_CO_2_ > 100 mmHg, pH < 7.1, K >6.0 mmol/l or apnoea-time > 30 minutes.

During the intervention, we will continuously measure arterial blood pressure, pulse oximetry, p_tc_CO_2_, p_tc_O_2_, heart rate, depth of anaesthesia (Narcotrend^TM^), brain oxygenation, cardiac output by pulse contour analysis, and ventilation distribution changes by thoracic electrical impedance tomography (EIT). Latter will be measured with a PulmoVista^®^ 500, by Dräger, Lübeck, Germany. Furthermore, we will take arterial blood samples at the onset of apnoea, one minute after apnoea, and thereafter every two minutes to perform blood gas analysis (total of max. 19 measurements).

During the experiment, one anaesthesia staff member will be free of all study related responsibilities in order to ensure the patient’s wellbeing during anaesthesia consisting of stable blood pressure, neuromuscular blockage, and anaesthesia depth.

The experiment stops, if one of the following criteria is met: SpO_2_<92%, p_tc_CO_2_ >100 mmHg, pH<7.1, K>6.0 mmol/l, t >30 minutes. Thereafter the airway will be secured in the planned manner and endexpiratory CO_2_ is measured. Another endexpiratory CO_2_ measurement will take place after a recruitment manoeuvre, before anaesthesia will continue without any further interruptions.

Patients will be visited before discharge on the first postoperative day or contacted by phone, and asked for any sort of pain (throat, mandibular joint, head, etc.), nausea, vomiting, nasal dryness, lip injuries, dental damages, other injuries, or any other discomfort or complication using a (modified) visual analogue scale (VAS), where 0 is no pain (or discomfort), and 10 is maximum pain (or discomfort) where applicable. We will also assess all kinds of injuries obtained during anaesthesia.

Overall, we will include 125 patients, 25 per group. Patients will be blinded as to their group allocations. Double-blinding is not possible, as devices differ from each other. Five patients of each of the four groups will be randomized to an apnoea-time of 30 minutes. Besides the extension of measuring time, the exclusion criteria will stay the same for these cohorts.

This design allows us to focus our study on the primary outcome, since we ensure, that free air circulation (and possible CO_2_ clearance) is possible by visually assessing airway patency. Through measurement of extended vital parameters, we can identify physiologic influence of rising CO_2_ in anaesthetized patients undergoing apnoeic oxygenation.

In addition, demographic data will be extracted from the AIS. This only includes standard data, that is collected through routine normal clinical workflow, such as age, sex, weight, height, BMI, airway risk factors, underlying diseases, smoking habits, ASA physical health status and indication for the surgery.

## Methods of minimising bias

### Randomisation

Patients will be assigned to their respective group using a computer-generated randomization list by www.randomisation.com. Randomisation will be stratified according to BMI in three groups: <25, 25-30 and >30 and smoker status (non-smoker, daily smokers < 40 years of age, daily smoker > 40 years of age and former smokers).

Allocated group will be kept in sealed opaque envelopes opened after induction of anaesthesia when proper face-mask ventilation is confirmed. Patients excluded from the study before opening of the randomisation envelope (e.g. because of impossible mask ventilation) will be replaced as if not eligible for the study.

### Blinding procedures

Patients are under general anaesthesia therefore blinded to the treatment group, but due to the different oxygen delivery systems study personnel in the operating theatre cannot be blinded.

## Unblinding Procedures (Code break)

Not needed, as this will be an open study.

# STUDY POPULATION

## Eligibility criteria

We will include consenting adult patients (≥ 18 years) undergoing elective surgery that requires general anaesthesia at the University Hospital Inselspital in Bern, ASA physical status 1-3 and written informed consent in German or French.

Exclusion criteria are:

- Any Indication for fibre optic intubation
- Expected impossible mask ventilation
- Known coronary heart disease
- Known heart failure, NYHA classification ≥ 2
- Arrhythmias in need of anti-arrhythmic therapy (e.g. implanted cardio defibrillator)
- Peripheral occlusive arterial disease, Fontaine ≥ 2b
- Known stenosis of the (common or internal) carotid or vertebral arteries
- Treatment with beta-receptor antagonists
- BMI > 35kg/m^2^ and BMI < 16kg/m^2^
- Hyperkalaemia (K > 5.5 mmol/l)
- Known COPD Gold classification ≥ 2
- Known pulmonary arterial hypertension, systolic > 35mmHg
- Known obstructive sleep apnoea syndrome in need of therapy
- High risk of aspiration (requiring rapid sequence induction intubation)
- Increased intracranial pressure
- Intracranial surgery
- Limited knowledge of German or French language
- Absent power of judgement
- Anaemia, Hb < 100 g/l
- Pregnancy (pregnancy test in all female patients)
- Neuromuscular disorder
- Known or suspected cervical spine instability
- Nasal obstruction, impossibility of nasal ventilation (both sides patent)
- Allergies or contra-indications to one or more of the used anaesthesia agents
- Age > 80 years

## Recruitment and screening

Patients are screened during the pre-anaesthesia visit before surgery. Eligible patients will be asked if they might participate in the study during the pre-anaesthesia visit and they will sign the informed consent. For female participants, we will additionally measure ß-HCG in the blood as part of the standard preoperative laboratory control, to rule out pregnancy.

## Assignment to study groups

Patients will be randomly assigned to their respective group using a randomization out of www.randomisation.com. Randomization will be stratified according to BMI (16-25kg/m^2^, 25-30 kg/m^2^, 30-35 kg/m^2^), smoker status (non-smoker, daily smoker, former smoker) and age (18-40 years, > 40 years).

## Criteria for withdrawal / discontinuation of participants

The study will be stopped if change of anaesthesia procedure (i.e. no general anaesthesia necessary) will be decided. Impossible mask ventilation before apnoea or any malfunction of necessary equipment (e.g. transcutaneous CO_2_ measurement) will also terminate the study.

# STUDY INTERVENTION

## Identity of Investigational treatment

### Experimental Intervention

**The first** group receives low-flow 2l/min of 100% humidified oxygen using Carbamed digiflow, Aquapak Hudson RCI and nasal cannula.

The **second** group receives 10 l/min of 100% humidified oxygen using Carbamed digiflow, Aquapak Hudson RCI and nasal cannula. The **third** group receives high-flow nasal cannula therapy with 70 l/min with 100% oxygen via OptiFlow™ by Fisher&Paykel™, Auckland, NewZealand, using jaw thrust. The **fourth** group gets intubated after the induction of the general anaesthesia. Then 0.25l/min 100% oxygen is delivered with the secured airway.

### Control Intervention (routine treatment)

**Control** group: High-flow nasal cannula therapy with 70 l/min with 100% oxygen via OptiFlow™ by Fisher&Paykel™, Auckland, NewZealand performing continuous laryngoscopy (standard of care), with a MacGrath^TM^ MAC videolaryngoscope, Medtronic, Dublin, Ireland.

## Administration of experimental and control interventions

### Experimental Intervention

After apnoea sets in and mask ventilation is successfully established, randomization envelopes are opened and according to group allocation, one of the following oxygen delivery systems is applied.

**The first** group receives low-flow 2l/min of 100% humidified oxygen using Carbamed digiflow, Aquapak Hudson RCI and nasal cannula.

The **second** group receives 10 l/min of 100% humidified oxygen using Carbamed digiflow, Aquapak Hudson RCI and nasal cannula.

The **third** group receives high-flow nasal cannula therapy with 70 l/min with 100% oxygen via OptiFlow™ by Fisher&Paykel™, Auckland, NewZealand, using jaw thrust.

The **fourth** group receives 0.25l/min of 100% oxygen with the secured airway.

### Control Intervention

Control group: High-flow nasal cannula therapy with 70 l/min with 100% oxygen via OptiFlow™ by Fisher&Paykel™, Auckland, NewZealand performing continuous laryngoscopy (standard of care), with a MacGrath^TM^ MAC videolaryngoscope, Medtronic, Dublin, Ireland.

## Data Collection and Follow-up for withdrawn participants

No further data collection after withdrawal, already collected data will be analyzed and after data evaluation anonymised. These patients will be visited after anaesthesia anyhow to ensure wellbeing.

## Study flow chart(s) / table of study procedures and assessments

| **Study procedures** | **Assessments** |
| --- | --- |
| High-flow nasal cannula therapy with 70 l/min with 100% oxygen, jaw thrust | Cerebral NIRS |
| High-flow nasal cannula therapy with 70 l/min with 100% oxygen, laryngoscopy | transcutaneous CO_2_ and O_2_ |
| Medium-flow nasal cannula therapy with 10 l/min with 100% oxygen | Time |
| Low-flow oxygen 100% through standard nasal cannula, 2 l/min | Saturation |
| Minimal-flow oxygen 100% via an endotracheal tube, 0.25ml/min | Post-medication interview |
|  | EIT |
|  | Narcotrend^TM^ |
|  | TOF |
|  | Arterial blood gas analyses |
|  | Airway patency using nasopharyngeal fiberscope |
|  | etCO_2_ |

## Assessments of outcomes

### Assessment of primary outcome

Transcutaneous CO_2_ will be measured throughout the study period by TCM4, Radiometer™, Thalwil, Switzerland and TCM5, Radiometer™, Thalwil, Switzerland; by applying a probe over the patient’s chest. This is a standard procedure used very often during anaesthesia without complications.

Increase in p_tc_CO_2_ in mmHg/min will be calculated by dividing the difference of p_tc_CO_2_ by the time of the measurement, once linear increase has set in. In our pilot study, we have found transcutaneous p_tc_CO_2_ to have a little delay (about 60-90 seconds) after which linear rise sets in.

### Assessment of secondary outcomes

Time to desaturation below 92% will be measured using a stopwatch. If desaturation does not set in, the experiment will end once 30 minutes have been completed.

Arterial blood pressure will be measured using a catheter in the radial artery. ECG and pulse oximetry will also be measured. These measurements are normal anaesthesia care.

From the arterial catheter blood samples will be drawn for blood gas analyses.

Brain oxygenation as a surrogate of brain perfusion will be assessed by cerebral NIRS, using Niro-200NX (Hamamatsu, Tokyo, Japan) monitor continuously. This is also a standard procedure without complications, especially during cardiac surgery.

EIT will be continuously measured using PulmoVista^®^ 500 by Dräger, Lübeck, Germany, to visualize possible atelectasis and its progression over time.
EIT is a non-invasive, radiation-free technique for the assessment of spatial and temporal ventilation distribution based on the changes in electrical properties of the tissue during the respiratory cycle. EIT measurements will be performed using a commercially available setup (PulmoVista 500, Draeger, Germany). A loose-fitting belt with 16 evenly spaced electrodes will be placed around the participant’s chest in thoracic median plane. Small electrical currents are injected through adjacent electrodes in a rotating mode. Resulting potential differences are measured, and impedance distribution sampled at 30 Hz will be calculated by an automated linearised newton-raphson reconstruction algorithm [19]. Relative change in end-expiratory lung impedance (EELI) and measures of ventilation inhomogeneity such as the global inhomogeneity index (GI) will be calculated as described previously using customized software (Matlab R2013a, The MathWorks Inc., Nattick, MA, USA) [20, 21].

After securing the airway, the highest etCO_2_ measured during the first minute after intubation and after a recruitment manoeuvre will be recorded.

### Assessment of safety outcomes

NIRS registers alterations in cerebral blood flow and perfusion. Arterial blood gas analysis will detect severe acidosis or hyperkalaemia.

#### Adverse events

Any adverse events during the study period will be recorded.

An **Adverse Event (AE)** is any untoward medical occurrence in a patient or a clinical investigation participant administered a pharmaceutical product, and which does not necessarily have a causal relationship with the study procedure. An AE can therefore be any unfavourable and unintended sign (including an abnormal laboratory finding), symptom, or disease temporally associated with the use of a medicinal (investigational) product, whether or not related to the medicinal (investigational) product. [ICH E6 1.2]

A **Serious Adverse Event (SAE)** is classified as any untoward medical occurrence that:

- results in death
- is life-threatening
- requires in-patient hospitalization or prolongation of existing hospitalisation
- results in persistent or significant disability/incapacity
- is a congenital anomaly/birth defect

In addition, important medical events that may not be immediately life-threatening or result in death, or require hospitalisation, but may jeopardise the patient or may require intervention to prevent one of the other outcomes listed above should also usually be considered serious. [ICH E2A].

All SAEs will be reported immediately and within a maximum of 24 hours to the Sponsor-Investigator of the study.

SAEs resulting in death are reported to the local Ethics Committee within 15 days.

SUSARs will be reported to the local Ethics Committee within 15 days.

#### **Laboratory parameters**

Arterial blood for blood gas analysis will be drawn awake, immediately after apnoea sets in, after one minute of apnoea and thereafter every two minutes. All in all, this makes ten blood gas analysis, for each of which we need to draw about 7.5 ml of blood. This is a study related measurement and not part of normal workflow.
Parameters analysed will include:

- pH
- p_a_CO_2_
- p_a_O_2_
- Saturation
- base excess
- bicarbonate
- potassium

#### **Vital signs**

During anaesthesia, standard monitoring of vital signs will be recorded, as defined by the American Society of Anesthesiologists and the Swiss Society for Anaesthesia and Resuscitation. This includes: EKG, end-tidal CO_2_, continuous arterial blood pressure through arterial catheter, pulse oximetry, body temperature.

## Procedures at each visit

First visit during pre-anaesthesia visit to rule out exclusion criteria and to obtain written informed consent and to complete any missing data from the pre-operative interview. A follow-up visit will be performed (structured post-medication interview) before discharge. This includes asking for possible side effects: sore throat/nose, mandibular pain, nausea, dental damages, oropharyngeal injuries of any kind, headaches as well as nasal dryness.

# SAFETY

Adverse events during the anaesthesia procedure will be treated according to current departmental guidelines at the discretion of the attending anaesthesia consultant.

Overall very little risk is involved for the patients, as apnoeic oxygenation has been shown to work in routine patient care.

The study can be terminated at any time points without delay and the airway can be secured immediately.

# STATISTICAL METHODS

## Hypothesis

Null hypothesis: The difference of the mean increase of p_tc_CO_2_ between any of the three intervention groups and the control group is at least 0.3 mmHg/min, the control group being lower.

Primary hypothesis: the difference of the mean increase of p_tc_CO_2_ between the groups is less than 0.3 mmHg/min (non-inferior margin), one-sided testing. This hypothesis was based on a small clinical pilot study that showed no differences regardless of flowrate or mouth opening (jaw-thrust vs. laryngoscopy).

Secondary outcome hypothesis: The number of patients not tolerating the apnoea time of 15 respectively 30 minutes is not significantly higher in any of the three intervention groups.

Secondary outcome hypothesis: no significant changes in vital parameters such as cerebral perfusion will be recorded.

## Determination of Sample Size

We assumed that with maximal flow of 70L O2/min and maximal mouth opening, there would be the least increase of p_tc_CO_2_/min. Patel et al. showed an increase of only 1.13mmHg using this technique [2]. However, this was etCO_2_ instead of p_tc_CO_2_. According to Gustafson et al., who measured p_tc_CO_2_ as well as p_a_CO_2_, the CO_2_ increase was in fact 1.8mmHg/min, with a standard deviation of 0.35mmHg [3]. This correlated very well with our own findings in our pilot study (not published).

Using a non-inferiority design, a difference of means of 0.3mmHg/min, a standard deviation of 0.35mmHg/min, an alpha of 0.025 (one-sided) and a power of 80%, the necessary sample size calculates to 22 patients per group. Therefore, we will include 25 patients in each group to account for possible drop outs. A value of 0.3mmHg/min would still only result in a difference in increase of 9mmHg in 30 minutes of apnoea time. We defined this as measurable, but still clinically acceptable, because the normal range of standard arterial pCO_2_ is 11mmHg (35-46 mmHg) [6].

## Planned Analyses

### Datasets to be analysed, analysis populations

Distribution of data will be checked using qq-plots and swilk command in stata, normal distributed data will be presented as means and standard deviation, otherwise median and interquartile range will be used. Proportions will be presented as numbers and percentage. Analytical statistics uses Mann-Whitney u-tests and Students t-test, according distribution, or ANOVA and Kruskal-Wallis for multiple groups. Each of the groups will a-priory be compared to the control group independently, without correction of multiple comparisons. Other multiple comparisons or subgroup-analyses will be performed using correction factors. Proportions will be compared by Chi-square tests or Fisher’s exact test. As sensitivity analyses, we will do regression analyses, using mixed effects linear regressions.

### Safety analysis

Incidences of side effects and other safety outcome variables will be compared with known results from the literature.

### Deviation(s) from the original statistical plan

Any deviation from the planned analyses will be listed and justified in the study report.

## Handling of missing data and drop-outs

Any missing data or drop-outs from will be listed and justified in the study report.

# QUALITY ASSURANCE AND CONTROL

## Data handling and record keeping / archiving

### Case Report Forms

Demographic data (age, BMI, sex, type of surgery) will be obtained from the departmental electronic anaesthesia information system AIS. Consent forms will be obtained at the pre-anaesthesia visit. Study data will be directly recorded in the CRF, which should also be considered being source data. These data will be transferred into the departmental research documentation system – REDCap, as this is routine in our research practice (see below).

### Record keeping / archiving

All study data will be archived for a minimum of 10 years after study termination or premature termination of the clinical trial.

Digital data obtained from monitors (e.g. Narcotrend, Lidco, Pulmovista, Nirs, TCM) will be transferred directly into the study data base.

All data will be transferred into a secure, web-based data integration platform (REDCap, Research Electronic Data Capture, Vanderbild University, 2004) and controlled independently by two members of the study team. Additionally, data are double-checked by comparing the screening records with the digital anaesthesia documenting system of the hospital.

## Data management

### Data Management System

At the research platform darwin (darwin.insel.ch) there are three locally separated dedicated servers installed for research data management. Host names are darwin0015 (productive instance), darwin0016 (backup instance) and darwin0017 (test instance). The servers are running on Windows Server and using SQL Server (Microsoft Corporation, One Microsoft Way, Redmond, WA 98052-7329 USA) for the relational database.

In order to comply with the federal act on research involving human being (HRA) REDCap (Research Electronic Data Capture, Vanderbild University, 2004) is the dedicated software application. The web application REDCap Server is implemented in Java and is running on the Apache Tomcat web server. The data are stored in relational database engines like PostgreSQL or Microsoft SQL server [22].

### Data security, access and back-up

REDCap Server provides web application security. REDCap Server can be configured for Secure Socket Layer (SSL) encrypted data transfer if needed.

The application has a group and role-based security model. Each user belongs to one or more security groups with specific sets of permissions in relation to folder or projects in the system. Only dedicated site administrators have access to the admin console enabling user management and changing security settings.

Data are stored and visualized in data grids either in the format of datasets, lists or assays. Each change of data is tracked and documented in corresponding audit log files.

The system can only be accessed entering a user name and password. All events are recorded in the user event list of the audit log files.

The application is running on a productive server with an image on a backup server. In addition, a regularly backup of the whole database is implemented using storage servers (darwinnas0001 and darwinnas0002) hosted at the darwin.insel.ch domain.

### Analysis and archiving

REDCap Server provides data analysis by integrated tools for creating filtered views and charts.

All data can be exported in different formats (excel, text, queries) suitable for transfer to a statistical software package of choice. In addition, data can be analysed creating R views.

All data will be archived and secured in the database as long as required by legislation.

### Electronic and central data validation

Data are being validated by the primary investigator and the sponsor when evaluating the results

## Monitoring

A study nurse who is not involved in the study otherwise, will control all data for its validity and completeness on a weekly basis. External monitoring will not occur, as this is an investigator-initiated study with no external funding.

# PUBLICATION AND DISSEMINATION POLICY

The results will be published in a peer-reviewed journal. Final decision on publishing the results will be kept by the project leaders. Authors of the publication will be team members who contributed to the design, conduct or analysis of the study and who approved of the final version of the manuscript.

# FUNDING

## Funding

This study is solely financed by a departmental research grant from the departmental anaesthesia research fund. There is no funding or financial influence through any other company. We will ask for sponsoring or reduced prices of FlowTrac sensors (Edward’s LifeScience) and of OptiFlow nasal cannulas (Fisher&Paykel).

# INSURANCE

Insurance will be provided by the Inselspital through Zürich-Versicherungs-Gesellschaft AG. Contract number: 15.369.591

# REFERENCES

[1. Cook, T.M., et al.,](http://flexikon.doccheck.com/de/Blutgasanalyse) *[Major complications of airway management in the UK: results of the Fourth National Audit Project of the Royal College of Anaesthetists and the Difficult Airway Society. Part 2: intensive care and emergency departments.](http://flexikon.doccheck.com/de/Blutgasanalyse)* [Br J Anaesth, 2011.](http://flexikon.doccheck.com/de/Blutgasanalyse) **[106](http://flexikon.doccheck.com/de/Blutgasanalyse)**[(5): p. 632-42.](http://flexikon.doccheck.com/de/Blutgasanalyse)

[2. Patel, A. and S.A. Nouraei,](http://flexikon.doccheck.com/de/Blutgasanalyse) *[Transnasal Humidified Rapid-Insufflation Ventilatory Exchange (THRIVE): a physiological method of increasing apnoea time in patients with difficult airways.](http://flexikon.doccheck.com/de/Blutgasanalyse)* [Anaesthesia, 2015.](http://flexikon.doccheck.com/de/Blutgasanalyse) **[70](http://flexikon.doccheck.com/de/Blutgasanalyse)**[(3): p. 323-9.](http://flexikon.doccheck.com/de/Blutgasanalyse)

[3. Gustafsson, I.M., et al.,](http://flexikon.doccheck.com/de/Blutgasanalyse) *[Apnoeic oxygenation in adults under general anaesthesia using Transnasal Humidified Rapid-Insufflation Ventilatory Exchange (THRIVE) - a physiological study.](http://flexikon.doccheck.com/de/Blutgasanalyse)* [Br J Anaesth, 2017.](http://flexikon.doccheck.com/de/Blutgasanalyse) **[118](http://flexikon.doccheck.com/de/Blutgasanalyse)**[(4): p. 610-617.](http://flexikon.doccheck.com/de/Blutgasanalyse)

[4. Frumin, M.J., R.M. Epstein, and G. Cohen,](http://flexikon.doccheck.com/de/Blutgasanalyse) *[Apneic oxygenation in man.](http://flexikon.doccheck.com/de/Blutgasanalyse)* [Anesthesiology, 1959.](http://flexikon.doccheck.com/de/Blutgasanalyse) **[20](http://flexikon.doccheck.com/de/Blutgasanalyse)**[: p. 789-98.](http://flexikon.doccheck.com/de/Blutgasanalyse)

[5. Eger, E.I. and J.W. Severinghaus,](http://flexikon.doccheck.com/de/Blutgasanalyse) *[The rate of rise of PaCO2 in the apneic anesthetized patient.](http://flexikon.doccheck.com/de/Blutgasanalyse)* [Anesthesiology, 1961.](http://flexikon.doccheck.com/de/Blutgasanalyse) **[22](http://flexikon.doccheck.com/de/Blutgasanalyse)**[: p. 419-25.](http://flexikon.doccheck.com/de/Blutgasanalyse)

[6. M. Habben, F.A.](http://flexikon.doccheck.com/de/Blutgasanalyse) *[Blutgasanalyse](http://flexikon.doccheck.com/de/Blutgasanalyse)*[. [Web Page] 2018 [cited 2018 19.1.2018]; Available from: http://flexikon.doccheck.com/de/Blutgasanalyse.](http://flexikon.doccheck.com/de/Blutgasanalyse)

[7. Teller, L.E., et al.,](http://flexikon.doccheck.com/de/Blutgasanalyse) *[Pharyngeal insufflation of oxygen prevents arterial desaturation during apnea.](http://flexikon.doccheck.com/de/Blutgasanalyse)* [Anesthesiology, 1988.](http://flexikon.doccheck.com/de/Blutgasanalyse) **[69](http://flexikon.doccheck.com/de/Blutgasanalyse)**[(6): p. 980-2.](http://flexikon.doccheck.com/de/Blutgasanalyse)

[8. Meltzer, S.J. and J. Auer,](http://flexikon.doccheck.com/de/Blutgasanalyse) *[Continuous Respiration without Respiratory Movements.](http://flexikon.doccheck.com/de/Blutgasanalyse)* [J Exp Med, 1909.](http://flexikon.doccheck.com/de/Blutgasanalyse) **[11](http://flexikon.doccheck.com/de/Blutgasanalyse)**[(4): p. 622-5.](http://flexikon.doccheck.com/de/Blutgasanalyse)

[9. Bartlett, R.G., Jr., H.F. Brubach, and H. Specht,](http://flexikon.doccheck.com/de/Blutgasanalyse) *[Demonstration of aventilatory mass flow during ventilation and apnea in man.](http://flexikon.doccheck.com/de/Blutgasanalyse)* [J Appl Physiol, 1959.](http://flexikon.doccheck.com/de/Blutgasanalyse) **[14](http://flexikon.doccheck.com/de/Blutgasanalyse)**[(1): p. 97-101.](http://flexikon.doccheck.com/de/Blutgasanalyse)

[10. Price, H.L.,](http://flexikon.doccheck.com/de/Blutgasanalyse) *[Effects of carbon dioxide on the cardiovascular system.](http://flexikon.doccheck.com/de/Blutgasanalyse)* [Anesthesiology, 1960.](http://flexikon.doccheck.com/de/Blutgasanalyse) **[21](http://flexikon.doccheck.com/de/Blutgasanalyse)**[: p. 652-63.](http://flexikon.doccheck.com/de/Blutgasanalyse)

[11. Sechzer, P.H., et al.,](http://flexikon.doccheck.com/de/Blutgasanalyse) *[Effect of carbon dioxide inhalation on arterial pressure, ECG and plasma catecholamines and 17-OH corticosteroids in normal man.](http://flexikon.doccheck.com/de/Blutgasanalyse)* [J Appl Physiol, 1960.](http://flexikon.doccheck.com/de/Blutgasanalyse) **[15](http://flexikon.doccheck.com/de/Blutgasanalyse)**[: p. 454-8.](http://flexikon.doccheck.com/de/Blutgasanalyse)

[12. Heard, A., et al.,](http://flexikon.doccheck.com/de/Blutgasanalyse) *[Apneic Oxygenation During Prolonged Laryngoscopy in Obese Patients: A Randomized, Controlled Trial of Buccal RAE Tube Oxygen Administration.](http://flexikon.doccheck.com/de/Blutgasanalyse)* [Anesth Analg, 2017.](http://flexikon.doccheck.com/de/Blutgasanalyse) **[124](http://flexikon.doccheck.com/de/Blutgasanalyse)**[(4): p. 1162-1167.](http://flexikon.doccheck.com/de/Blutgasanalyse)

[13. Lyons, C. and M. Callaghan,](http://flexikon.doccheck.com/de/Blutgasanalyse) *[Apnoeic oxygenation with high-flow nasal oxygen for laryngeal surgery: a case series.](http://flexikon.doccheck.com/de/Blutgasanalyse)* [Anaesthesia, 2017.](http://flexikon.doccheck.com/de/Blutgasanalyse) **[72](http://flexikon.doccheck.com/de/Blutgasanalyse)**[(11): p. 1379-1387.](http://flexikon.doccheck.com/de/Blutgasanalyse)

[14. Bain, A.R., et al.,](http://flexikon.doccheck.com/de/Blutgasanalyse) *[Cerebral oxidative metabolism is decreased with extreme apnoea in humans; impact of hypercapnia.](http://flexikon.doccheck.com/de/Blutgasanalyse)* [J Physiol, 2016.](http://flexikon.doccheck.com/de/Blutgasanalyse) **[594](http://flexikon.doccheck.com/de/Blutgasanalyse)**[(18): p. 5317-28.](http://flexikon.doccheck.com/de/Blutgasanalyse)

[15. Miyamoto, K. and M. Nishimura,](http://flexikon.doccheck.com/de/Blutgasanalyse) *[Nasal dryness discomfort in individuals receiving dry oxygen via nasal cannula.](http://flexikon.doccheck.com/de/Blutgasanalyse)* [Respir Care, 2008.](http://flexikon.doccheck.com/de/Blutgasanalyse) **[53](http://flexikon.doccheck.com/de/Blutgasanalyse)**[(4): p. 503-4.](http://flexikon.doccheck.com/de/Blutgasanalyse)

[16. Dellweg, D., et al.,](http://flexikon.doccheck.com/de/Blutgasanalyse) *[Humidification of inspired oxygen is increased with pre-nasal cannula, compared to intranasal cannula.](http://flexikon.doccheck.com/de/Blutgasanalyse)* [Respir Care, 2013.](http://flexikon.doccheck.com/de/Blutgasanalyse) **[58](http://flexikon.doccheck.com/de/Blutgasanalyse)**[(8): p. 1323-8.](http://flexikon.doccheck.com/de/Blutgasanalyse)

[17. Scheer, B.V., A. Perel, and U.J. Pfeiffer,](http://flexikon.doccheck.com/de/Blutgasanalyse) *[Clinical review: Complications and risk factors of peripheral arterial catheters used for haemodynamic monitoring in anaesthesia and intensive care medicine.](http://flexikon.doccheck.com/de/Blutgasanalyse)* [Crit Care, 2002.](http://flexikon.doccheck.com/de/Blutgasanalyse) **[6](http://flexikon.doccheck.com/de/Blutgasanalyse)**[(3): p. 199-204.](http://flexikon.doccheck.com/de/Blutgasanalyse)

[18. Uzun, L., et al.,](http://flexikon.doccheck.com/de/Blutgasanalyse) *[Effectiveness of the jaw-thrust maneuver in opening the airway: a flexible fiberoptic endoscopic study.](http://flexikon.doccheck.com/de/Blutgasanalyse)* [ORL J Otorhinolaryngol Relat Spec, 2005.](http://flexikon.doccheck.com/de/Blutgasanalyse) **[67](http://flexikon.doccheck.com/de/Blutgasanalyse)**[(1): p. 39-44.](http://flexikon.doccheck.com/de/Blutgasanalyse)

[19. Yorkey, T.J., J.G. Webster, and W.J. Tompkins,](http://flexikon.doccheck.com/de/Blutgasanalyse) *[Comparing reconstruction algorithms for electrical impedance tomography.](http://flexikon.doccheck.com/de/Blutgasanalyse)* [IEEE Trans Biomed Eng, 1987.](http://flexikon.doccheck.com/de/Blutgasanalyse) **[34](http://flexikon.doccheck.com/de/Blutgasanalyse)**[(11): p. 843-52.](http://flexikon.doccheck.com/de/Blutgasanalyse)

[20. Schnidrig, S., et al.,](http://flexikon.doccheck.com/de/Blutgasanalyse) *[Influence of end-expiratory level and tidal volume on gravitational ventilation distribution during tidal breathing in healthy adults.](http://flexikon.doccheck.com/de/Blutgasanalyse)* [Eur J Appl Physiol, 2013.](http://flexikon.doccheck.com/de/Blutgasanalyse) **[113](http://flexikon.doccheck.com/de/Blutgasanalyse)**[(3): p. 591-8.](http://flexikon.doccheck.com/de/Blutgasanalyse)

[21. Wettstein, M., L. Radlinger, and T. Riedel,](http://flexikon.doccheck.com/de/Blutgasanalyse) *[Effect of different breathing aids on ventilation distribution in adults with cystic fibrosis.](http://flexikon.doccheck.com/de/Blutgasanalyse)* [PLoS One, 2014.](http://flexikon.doccheck.com/de/Blutgasanalyse) **[9](http://flexikon.doccheck.com/de/Blutgasanalyse)**[(9): p. e106591.](http://flexikon.doccheck.com/de/Blutgasanalyse)

[22. Nelson, E.K., et al.,](http://flexikon.doccheck.com/de/Blutgasanalyse) *[LabKey Server: an open source platform for scientific data integration, analysis and collaboration.](http://flexikon.doccheck.com/de/Blutgasanalyse)* [BMC Bioinformatics, 2011.](http://flexikon.doccheck.com/de/Blutgasanalyse) **[12](http://flexikon.doccheck.com/de/Blutgasanalyse)**[: p. 71.](http://flexikon.doccheck.com/de/Blutgasanalyse)
